# Leaf Treatments with a Protein-Based Resistance Inducer Partially Modify Phyllosphere Microbial Communities of Grapevine

**DOI:** 10.3389/fpls.2016.01053

**Published:** 2016-07-19

**Authors:** Martina Cappelletti, Michele Perazzolli, Livio Antonielli, Andrea Nesler, Esmeralda Torboli, Pier L. Bianchedi, Massimo Pindo, Gerardo Puopolo, Ilaria Pertot

**Affiliations:** ^1^Department of Sustainable Ecosystems and Bioresources, Research and Innovation Centre, Fondazione Edmund MachSan Michele all'Adige, Italy; ^2^Department of Agricultural and Environmental Sciences, University of UdineUdine, Italy; ^3^Bioresources Unit, Department of Health and Environment, Austrian Institute of TechnologyTulln and der Donau, Austria; ^4^Technology Transfer Center, Fondazione Edmund MachSan Michele all'Adige, Italy

**Keywords:** resistance induction, gene expression, *Vitis vinifera*, *Plasmopara viticola*, phyllosphere microbiota, biological control

## Abstract

Protein derivatives and carbohydrates can stimulate plant growth, increase stress tolerance, and activate plant defense mechanisms. However, these molecules can also act as a nutritional substrate for microbial communities living on the plant phyllosphere and possibly affect their biocontrol activity against pathogens. We investigated the mechanisms of action of a protein derivative (nutrient broth, NB) against grapevine downy mildew, specifically focusing on the effects of foliar treatments on plant defense stimulation and on the composition and biocontrol features of the phyllosphere microbial populations. NB reduced downy mildew symptoms and induced the expression of defense-related genes in greenhouse- and *in vitro*-grown plants, indicating the activation of grapevine resistance mechanisms. Furthermore, NB increased the number of culturable phyllosphere bacteria and altered the composition of bacterial and fungal populations on leaves of greenhouse-grown plants. Although, NB-induced changes on microbial populations were affected by the structure of indigenous communities originally residing on grapevine leaves, degrees of disease reduction and defense gene modulation were consistent among the experiments. Thus, modifications in the structure of phyllosphere populations caused by NB application could partially contribute to downy mildew control by competition for space or other biocontrol strategies. Particularly, changes in the abundance of phyllosphere microorganisms may provide a contribution to resistance induction, partially affecting the hormone-mediated signaling pathways involved. Modifying phyllosphere populations by increasing natural biocontrol agents with the application of selected nutritional factors can open new opportunities in terms of sustainable plant protection strategies.

## Introduction

Increasing concerns about the negative impacts of chemical pesticides on human health and the environment require the development of safe alternatives to conventional disease control methods (Fantke et al., 2012). Elicitors, including beneficial microorganisms and exogenous molecules of both biological and synthetic origin, can stimulate plant defenses against pathogens (Boller and Felix, 2009; Walters et al., 2013), and they represent some of the most promising complementary/alternative strategies to reduce the massive use of fungicides (Delaunois et al., 2014). The mechanism of action of these compounds relies on the rapid activation of sophisticated defense systems after perception by plant cells, leading to specific transcriptional and metabolic modulations, such as the up-regulation of genes encoding pathogenesis related (PR) proteins (Wu et al., 2014). Two main pathways are known to be activated by elicitors: the systemic acquired resistance that is mediated by salicylic acid (SA)-dependent processes, and the induced systemic resistance that is mediated by jasmonic acid (JA)- and ethylene-regulated pathways (Pieterse et al., 2009; Walters et al., 2013).

Because grapevine (*Vitis vinifera* L.) is a major fruit crop worldwide, it represents an ideal model for studying interactions between the plant, elicitor and pathogen. Commercial grapevine cultivars are highly susceptible to a destructive disease (namely downy mildew) caused by the obligate parasite *Plasmopara viticola* (Berk. and Curt.) Berl. and de Toni, and require frequent fungicide applications to avoid yield and quality losses (Gessler et al., 2011). Several elicitors are known to activate defense responses against grapevine downy mildew, including *Solidago canadensis* extracts (Harm et al., 2011), organic amendments (Thuerig et al., 2011), and fungi such as *Trichoderma harzianum* T39 (Perazzolli et al., 2008), or *Aureobasidium pullulans* (Harm et al., 2011). Likewise, the application of fosetyl-aluminum (Dercks and Creasy, 1989), ß-aminobutyric acid (Hamiduzzaman et al., 2005), and benzothiadiazole-7-carbothioic acid S-methyl ester (Perazzolli et al., 2008) has been shown to induce grapevine resistance mechanisms. Bacterial proteins, such as harpin and flagellin (Qiao et al., 2010; Chang and Nick, 2012; Trdà et al., 2014), oligosaccharides, as oligogalacturonide (Allegre et al., 2009), and vitamins, such as thiamine and riboflavin (Boubakri et al., 2012, 2013), have been demonstrated to elicit grapevine defense responses. Resistance induction to downy mildew in grapevine includes primarily the up-regulation of defense-related genes, such as genes encoding PR-1, PR-2, PR-4, chitinase 3 (CHIT-3), and osmotin (OSM-1 and OSM-2) proteins (Perazzolli et al., 2011, 2012).

Generally speaking, carbohydrates and proteins or peptides represent a wide category of plant resistance inducers (Albert, 2013; Trouvelot et al., 2014). Protein hydrolysates consist of a mixture of small peptide fragments and free amino acids, originating from animal, plant and microbial proteins by chemical or enzymatic hydrolysis, which could act as biostimulants by influencing the growth process or by directly stimulating plant defense responses (Colla et al., 2015). For example, some plant-derived protein hydrolysates have been shown to increase the activity of the plant antioxidant system and others have demonstrated beneficial effects on plant metabolism, including shoot and root growth (Colla et al., 2015). Recently, casein and soybean hydrolysates have been shown to act as elicitors of grapevine defense mechanisms against downy mildew and gray mold by the up-regulation of *PR* genes (Lachhab et al., 2014, 2016). Likewise, a protein derivative (nutrient broth, NB) showed a high efficacy in controlling powdery mildew in vineyards by inducing the expression of defense-related genes which demonstrate stimulation of plant defense mechanisms (Nesler et al., 2015). Similarly, increasing interest has been devoted to the use of carbohydrates to stimulate plant resistance against diseases, either as elicitors of plant defenses or signaling molecules that mimic phytohormones (Trouvelot et al., 2014). For instance, chitin, chitosan, oligogalacturonides, and storage polysaccharides, such as ß-1-3 glucans extracted from the brown alga *Laminaria digitata* (namely laminarin), have been reported to stimulate plant defense reactions against several phytopathogens (Trouvelot et al., 2014). In grapevine, chitosan (Aziz et al., 2006), laminarin (Aziz et al., 2003), sulfated laminarin (Trouvelot et al., 2008), β-glucans and oligogalacturonides (Allegre et al., 2009) act as resistance inducers against *P. viticola*. In addition to plant resistance activation, protein hydrolysates (Colla et al., 2015) and carbohydrates (Trouvelot et al., 2014) can also serve as nutritional sources for microbial phyllosphere communities. Indeed, plant leaves are colonized by complex microbial communities, whose structure can be affected by environmental (such as UV radiation, pollution, and nitrogen fertilization) and biotic (such as leaf age and invading microorganisms) factors (Vorholt, 2012). When protein- or carbohydrate-based treatments are applied on leaves, they may change the nutrient availability, exerting a selective pressure on structure, dynamics, and functional properties of phyllosphere communities. Phyllosphere microbial communities frequently show positive influences on plant health and growth (Peñuelas and Terradas, 2014). For example, some phyllosphere microorganisms are regarded as natural biological control agents, thanks to their ability to reinforce natural plant defenses and to their antagonism to pathogens (Vorholt, 2012; Ritpitakphong et al., 2016) through the production of antimicrobial compounds, competition for space and nutrients, parasitism, or by combinations of these mechanisms (Pal and McSpadden Gardener, 2006).

The aim of this research was to understand whether the efficacy of NB against pathogens is related only to induction of resistance on grapevine or also to an indirect effect from modifications of leaf microbial communities. Grapevine downy mildew was selected as the study pathosystem. The effect of NB foliar application was assessed on plants in the absence (axenic conditions) and in the presence of phyllosphere microorganisms (greenhouse conditions), and its impact on natural grapevine phyllosphere microbiome was evaluated with both culture dependent and independent approaches in comparison to the application of a laminarin-based product (LAM) as a reference for resistance induction.

## Materials and methods

### Grapevine treatments and pathogen inoculation *in vitro*

Grapevine rooted cuttings (Pinot noir ENTAV115) were grown *in vitro* on Murashige-Skoog medium half dose with 3% sucrose and 0.6% agarose in De Wit cultures tubes (Duchefa Biochemie, Haarlem, The Netherlands) for 1 month in a growth chamber at 23 ± 1°C with a photoperiod of 16 h of light. Plants were treated with sterilized water (H_2_O) or with a sterilized solution of 3.0 g/l NB. NB was obtained by mixing three commercial extracts commonly used as nutritional substrates in microbiological media: 0.4 g/l meat extract (product code 70164, Fluka, Sigma-Aldrich, St. Louis, MO, USA), 0.7 g/l yeast extract (product code 70161, Fluka, Sigma-Aldrich) and 1.9 g/l peptone (product code 70175, Fluka, Sigma-Aldrich), and this application dosage was previously optimized against grapevine powdery mildew (Nesler et al., 2015).

Each leaf of *in vitro*-grown plants was treated with six to eight drops (20 μl each) of H_2_O or NB on the abaxial and adaxial surface and plants were incubated for 3 days in the growth chamber to maximize the phenotypic response of grapevine induced resistance (Perazzolli et al., 2008; Nesler et al., 2015). Each leaf was then dried with a sterile filter paper under sterile conditions and immediately inoculated with a sterile suspension of *P. viticola* (4 × 10^4^ sporangia/ml) as described by Algarra Alarcon et al. (2015) and the disease severity was assessed visually as percentage of leaf area covered by sporulation after 7 days (EPPO, 2001). For gene expression analyses, samples were collected in triplicates just before (T0) and 1 day after (T1) *P. viticola* inoculation. This time point was chosen because it is associated with leaf colonization by primary hyphae (Lenzi et al., 2015) and with modulation of defense-related genes for the establishment of resistance responses (Hamiduzzaman et al., 2005; Trouvelot et al., 2008; Polesani et al., 2010; Perazzolli et al., 2012). Each sample comprised two leaves from the second-fourth node of one plant. Six plants were analyzed for each treatment in a randomized complete block design and the experiment was carried out twice.

### Grapevine treatments and pathogen inoculation under greenhouse conditions

Two-year-old plants of the susceptible grapevine cultivar Pinot noir ENTAV115 grafted onto Kober 5BB were grown for 2 months under greenhouse conditions as described by Perazzolli et al. (2012). Plants were kept untreated (UNT) or treated with H_2_O, 3.0 g/l of NB, or 0.75 ml/l of a laminarin-based commercial product (LAM, dosage according to the manufacturer's instruction of Vacciplant, Belchim Crop Protection, Londerzeel, Belgium) used as a reference of resistance inducers from natural origin in grapevine (Aziz et al., 2003). Treatments were applied for three consecutive days (1, 2, and 3 days before *P. viticola* inoculation), in order to maximize the phenotypic response of grapevine induced resistance (Perazzolli et al., 2008; Nesler et al., 2015). One day after the last treatment, plants were inoculated as described by Perazzolli et al. (2012), and the disease severity was assessed visually after 7 days (EPPO, 2001). The disease reduction (efficacy) was calculated according to the following formula: (disease severity of H_2_O-treated plants—disease severity in plants treated with a tested molecule)/(disease severity of H_2_O-treated plants) × 100. Three replicates (pool of two plants each) were collected just before (T0) and 1 day after (T1) *P. viticola* inoculation for each treatment. Each sample comprised four half-leaves (collected from the fourth-sixth node of two plants) and 50 leaves (randomly collected from two plants) for the gene expression and microbial community analysis, respectively. Twelve plants were analyzed for each treatment in a randomized complete block design, and the experiment was carried out twice (namely Exp 1 and Exp 2). Under greenhouse conditions, UNT samples were used to compare indigenous microbial populations originally residing on grapevine leaves in the two different experiments, while effects of treatments tested were evaluated considering H_2_O-treated plants as reference control at each time point.

### RNA extraction and gene expression analyses

Total RNA extraction, DNase treatment, cDNA synthesis, and quantitative real-time PCR (qPCR) reactions were carried out as previously described (Lenzi et al., 2015) using specific primers (Table S1). Cycle threshold values and reaction efficiencies were calculated with the LightCycler 480 SV1.5.0 software (Roche, Branford, CT, USA) and the LinRegPCR 11.1 software (Ruijter et al., 2009), respectively. Relative expression levels of each gene were calculated with the Pfaffl equation (Pfaffl, 2001) on three replicates and two independent experiments, using the grapevine *Actin* gene for normalization (Polesani et al., 2010; Perazzolli et al., 2012).

### Isolation of grapevine phyllosphere microorganisms

Phyllosphere microorganisms were collected by leaf washing as described by Perazzolli et al. (2014). Each sample was plated on Nutrient Agar supplemented with 100 mg/l cycloheximide and on Potato Dextrose Agar supplemented with 0.25% lactic acid to isolate culturable bacteria and fungi, respectively. Plates were incubated at 25°C for 48 h, the colony forming units (CFU) per unit of leaf area (CFU/cm2) were calculated, and representative isolates were selected visually for each treatment and experiment based on morphological analysis of bacterial colonies.

### Functional characterization of culturable phyllosphere bacteria

Proteolytic activity, siderophore production and antagonist activity against *Phytophthora infestans* were evaluated for culturable bacteria as described by Puopolo et al. (2010). Three replicates were analyzed for each bacterial isolate and each assay was carried out twice.

For the assay against *P. viticola*, each bacterial isolate was grown in 1 ml of Luria Bertani medium under orbital shaking at 80 rpm at 27°C for 24 h. The bacterial suspension was centrifuged (5000 g for 15 min), washed three times in 1 ml of isotonic solution (NaCl 0.85%), and adjusted to an optical density of 0.2 at 600 nm. Each bacterial suspension was mixed with an equal volume of a sterile suspension of *P. viticola* sporangia (2 × 10^4^ sporangia/ml). Surface-sterilized leaf disks were prepared according to Perazzolli et al. (2014), inoculated with three 10 μl-drops of inoculum suspension for each disk and incubated overnight in the dark at 25 ± 1°C. Disks were dried under laminar flow and incubated under greenhouse conditions for 7 days before visual assessment of disease severity (EPPO, 2001). Five replicates (five dishes with five leaf disks each) were analyzed for each bacterial isolate and the experiment was carried out twice. The two bacterial isolates with biocontrol activity against *P. viticola* were identified by amplification of the V6-V8 region of the 16S rRNA by colony PCR, followed by sequencing with an ABI PRISM 3730xl DNA analyzer (Applied Biosystems, Thermo Fisher Scientific, Waltham, MA, USA) and alignment against the database of the National Center for Biotechnology Information (NCBI; http://www.ncbi.nlm.nih.gov). Sequences were deposited at the Sequence Read Archive of NCBI (http://www.ncbi.nlm.nih.gov/sra) under the accession numbers KU596386 (*Pseudomonas* spp. isolate T1_NB_7 of Exp 1) and KU596387 (*Enterobacter* spp. isolate T1_NB_13 of Exp 2).

### DNA extraction, amplification, and pyrosequencing

Microbial pellets were obtained from leaf-washing suspensions as described by Perazzolli et al. (2014), and DNA was extracted from microbial pellets using the FastDNA SPIN Kit for Soil (MP Biomedicals, Santa Ana, USA). Bacterial sequences were amplified with primer pairs (Pinto et al., 2014) that amplify the V6-V8 region of the 16S rRNA (Baker et al., 2003), and fungal sequences were amplified with primer pairs that align to the ITS3 and ITS4 regions of the internal transcribed spacer (ITS) fragment (White et al., 1990). Fusion primers with the Lib-L Primer sequences for unidirectional pyrosequencing (Roche) were used (Table S2), and amplicons were obtained from 100 ng of extracted DNA, using the FastStart High-Fidelity PCR system (Roche) with 0.25 mM deoxynucleoside triphosphates, 1% (w/v) bovine serum albumin, 4% (v/v) dimethyl sulfoxide, 0.3 μM of each primer, and 2.5 U of FastStart High-Fidelity DNA polymerase (Roche) in 50 μl of reaction. Amplification reactions were carried out in triplicate with the following protocol: denaturation at 95°C for 5 min, 32 cycles of amplification at 95°C for 30 s, annealing at 60 and 58°C for 1 min for bacteria and fungi, respectively, extension at 72°C for 45 s, and final extension at 72°C for 10 min. No amplification of 16S and ITS fragments was obtained from leaf-washing suspensions of *in vitro*-propagated plants, confirming that these plants were grown under axenic conditions.

Library construction and pyrosequencing were carried out as described by Perazzolli et al. (2014). Briefly, PCR products were purified using an AMPure XP bead kit (Beckman Coulter, Brea, CA, USA), quantified using a Roche 454 Titanium library quantification kit (KAPA Biosystems, Boston, MA, USA) and pyrosequenced using a GS FLX+ system (Roche) with the XL+ chemistry (Roche). Sequences have been deposited at the Sequence Read Archive of NCBI (http://www.ncbi.nlm.nih.gov/sra) under the accession number SRP065898 and BioProject number PRJNA301108.

### Bioinformatics analysis and 16S rRNA gene and its sequence processing

Bacterial and fungal sequences were processes as reported by Touceda-Gonzalez et al. (2015) with some modifications. Sequence quality check and filtering were carried out with PRINSEQ (Schmieder and Edwards, 2011) (http://prinseq.sourceforge.net/) and FlowClus (https://github.com/jsh58/FlowClus), respectively. For quality filtering, reads shorter than 150 bases or longer than 1000 bases were discarded, and homopolymer runs longer than six bases were excluded, as well as ambiguous sequences longer than six bases. A Phred quality score greater than 25 in a sliding window of 50 bases was considered as the minimum average allowed, and one barcode correction and two primer mismatches were accepted. Quality filtered reads were processed using V-Xtractor (Hartmann et al., 2010) (http://www.microbiome.ch/Tools.html) and ITSx (Bengtsson-Palme et al., 2013) (http://microbiology.se/software/itsx), in order to obtain highly reliable 16S V6-V8 rRNA and ITS2 sequences, respectively. USEARCH v7 (Edgar, 2013) (http://www.drive5.com) was used to de-replicate and sort the extracted regions. Chimeras were removed with UCHIME (Edgar et al., 2011) (http://drive5.com/usearch/manual/uchime_algo.html) using the ChimeraSlayer's database (http://microbiomeutil.sourceforge.net/#A_CS) (Haas et al., 2011) and the UNITE reference sequences (Koljalg et al., 2013) for bacterial and fungal sequences, respectively.

Clustering of operational taxonomic units (OTU) was carried out using the USEARCH v7 tool with 97% of pairwise sequence identity (Edgar, 2013). QIIME (Caporaso et al., 2010) (http://qiime.org) was used for taxonomy assignments of bacterial and fungal OTU with a naïve Bayesian RDP classifier and a minimum confidence of 0.8 (Wang et al., 2007) against the Greengenes database (August, 2013) (http://greengenes.secondgenome.com and UNITE database) (March, 2015) (http://www2.dpes.gu.se/project/unite/UNITE_intro.htm), respectively. After taxonomic classification in the Greengenes database, OTU corresponding to chloroplasts and mitochondrial sequences were discarded. The percentage of the total OTU that were sequenced in each sample was estimated using the Good's coverage estimator (Good, 1953).

### Statistical analysis

Statistical analysis of bacterial and fungal data were carried out as reported by Touceda-Gonzalez et al. (2015) with some modifications. The BIOM table generated by the 16S rRNA gene and ITS analysis was subsampled via multiple rarefaction in QIIME (Caporaso et al., 2010). For alpha-diversity metrics, the Chao1 index (Chao, 1984) and the Simpson's diversity index (Simpson, 1949) were calculated to estimate OTU richness and microbial diversity, respectively. For beta-diversity metrics, the BIOM table was processed with the metagenomeSeq Bioconductor package (Paulson et al., 2013; McMurdie and Holmes, 2014) (https://bioconductor.org/packages/release/bioc/html/metagenomeSeq.html) and a multivariate analysis was performed with an unsupervised Principal Component Analysis (data not shown) followed by its constrained ordination counterpart, i.e., a Canonical Analysis of Principal coordinates (CAP) (Anderson and Willis, 2003), a permutation test (Legendre and Legendre, 1998), and a permutational multivariate analysis of variance (PERMANOVA) (Anderson, 2001) implemented in the vegan R package (https://cran.r-project.org/web/packages/vegan/index.html). Significant differences among communities were assessed using the Bray-Curtis dissimilarity distance (Bray and Curtis, 1957) and ordination analyses were carried out with the phyloseq R package (McMurdie and Holmes, 2013) (https://joey711.github.io/phyloseq). When significant (*P* < 0.05) differences of treatment and/or experiment were detected, pairwise comparisons between treatments were carried out by the RVAideMemoire package with false discovery rate corrections for multiple testing (https://cran.r-project.org/web/packages/RVAideMemoire/index.html).

Data on disease severity, observed species, Chao1 and Simpson indexes, microbial relative abundances, and gene expression levels were processed using Statistica 9 Software (StatSoft, Tulsa, OK, USA). Three replicates (namely A, B, C) were analyzed for each treatment and each time point in two independent experiments (Exp 1 and Exp 2). Relative abundances of bacteria and fungi were normalized by arcsine transformation. Disease severity scores, CFU counts, and fold change values were transformed by square root, Log_10_, and the equation y = Log_10_ (1 + x) (Casagrande et al., 2011), respectively. When the *F*-test demonstrated non-significant treatment-experiment interactions (α > 0.05), data from the two experiments were pooled. After validating data for normal distribution (K-S test, *P* > 0.05) and variance homogeny (Cochran's test, *P* > 0.05), analysis of variance (one-way ANOVA) was carried out using Fisher's test (α = 0.05) to reveal significant differences among treatments and time points.

## Results

### Effects of nutrient broth against downy mildew under axenic conditions

**Figure 1:**
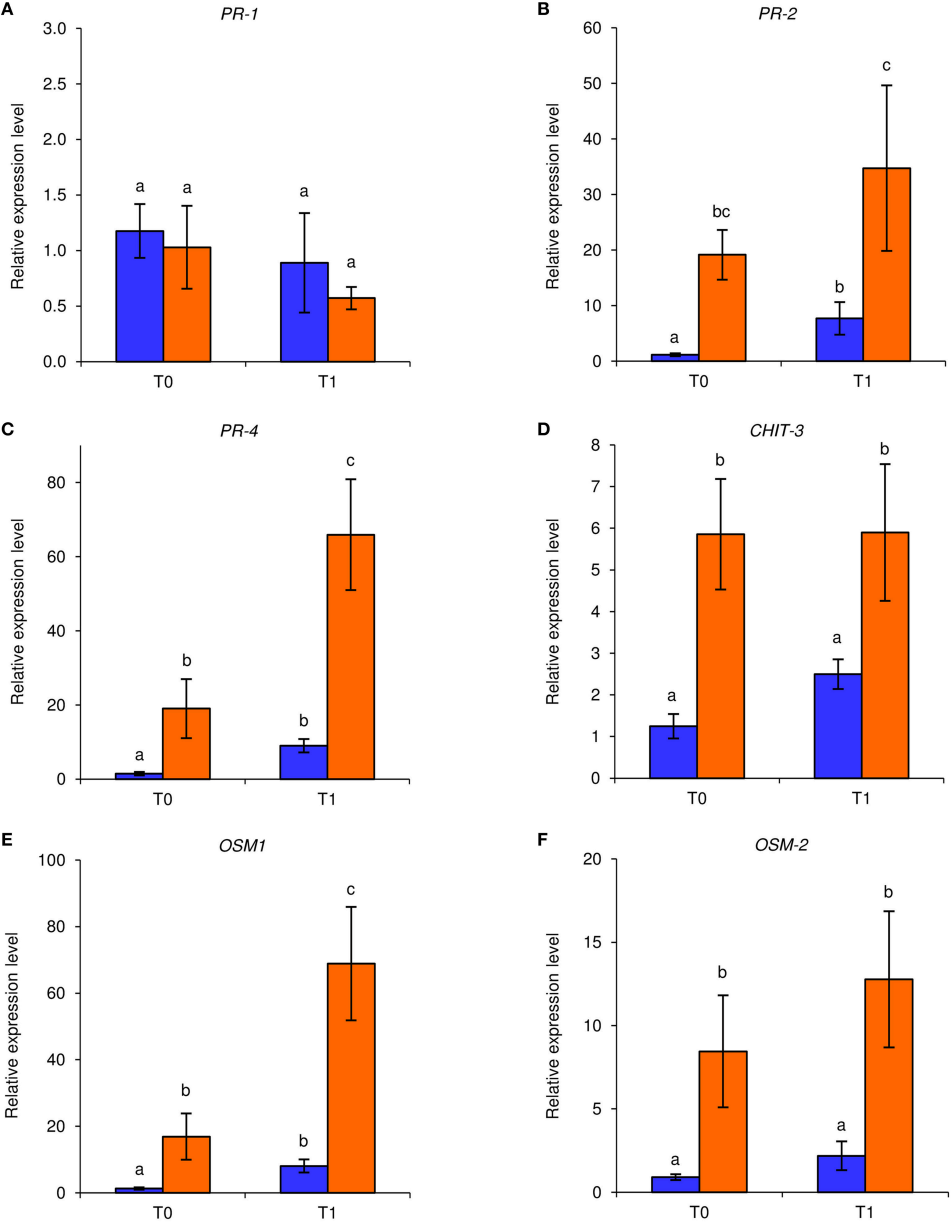
**Effect of leaf treatments on gene expression of grapevine plants under axenic conditions**. *In vitro*-grown grapevines were treated with water (blue) or nutrient broth (orange) for three consecutive days before pathogen inoculation. Leaf samples were collected just before (T0) and 1 day after (T1) inoculation with *Plasmopara viticola*. Relative expression levels of genes encoding the pathogenesis-related (PR) protein 1 (*PR-1*; **A**), *PR-2* **(B)**, *PR-4* **(C)**, chitinase 3 (*CHIT-3*; **D)**, osmotin 1 (*OSM-1*; **E**), and *OSM-2* **(F)** were assessed by quantitative real-time PCR. Relative expression levels were calculated using *Actin* as constitutive gene for normalization, and data were calibrated on water-treated plants at T0. An *F*-test revealed non-significant differences between experiments (*P*-values ranged from 0.06 to 0.91 for the genes tested), and data from the two experiments were pooled. Mean levels of relative expression and standard errors of six replicates (plants) pooled from two experiments are presented for each treatment and time point. For each gene, different letters indicate significant differences according to Fisher's test (α = 0.05).

Under axenic conditions, foliar applications of NB reduced downy mildew symptoms on *in vitro*-grown grapevines in two independent experiments. An *F*-test demonstrated non-significant effect of the experiment (*P* = 0.79), and data were pooled. Disease severity was significantly lower (Fisher test; α = 0.05) in NB-treated (disease severity: 1.2 ± 0.9%; average ± standard error) with respect to H_2_O-treated plants (disease severity: 19.1 ± 5.6%). In order to investigate the molecular mechanisms induced by NB in grapevine, expression levels of six defense-related genes (Table S1) were analyzed by qPCR in leaves collected at T0 and T1 of *P. viticola* inoculation (Figure 1). Under these axenic conditions, the expression of *PR-2, PR-4, CHIT-3, OSM-1*, and *OSM-2* was induced by NB at T0 and it remained at a high level at T1. In particular, expression of *PR-4* and *OSM-1* was further enhanced by *P. viticola* inoculation in NB-treated plants and expression of *PR-2, PR-4, CHIT-3, OSM-1*, and *OSM-2* was higher in NB-treated plants in comparison to H_2_O-treated plants at T1. Conversely, expression of *PR-1* was not affected by NB treatment or by *P. viticola* inoculation under axenic conditions.

### Assessment of grapevine resistance against downy mildew under greenhouse conditions

**Figure 2:**
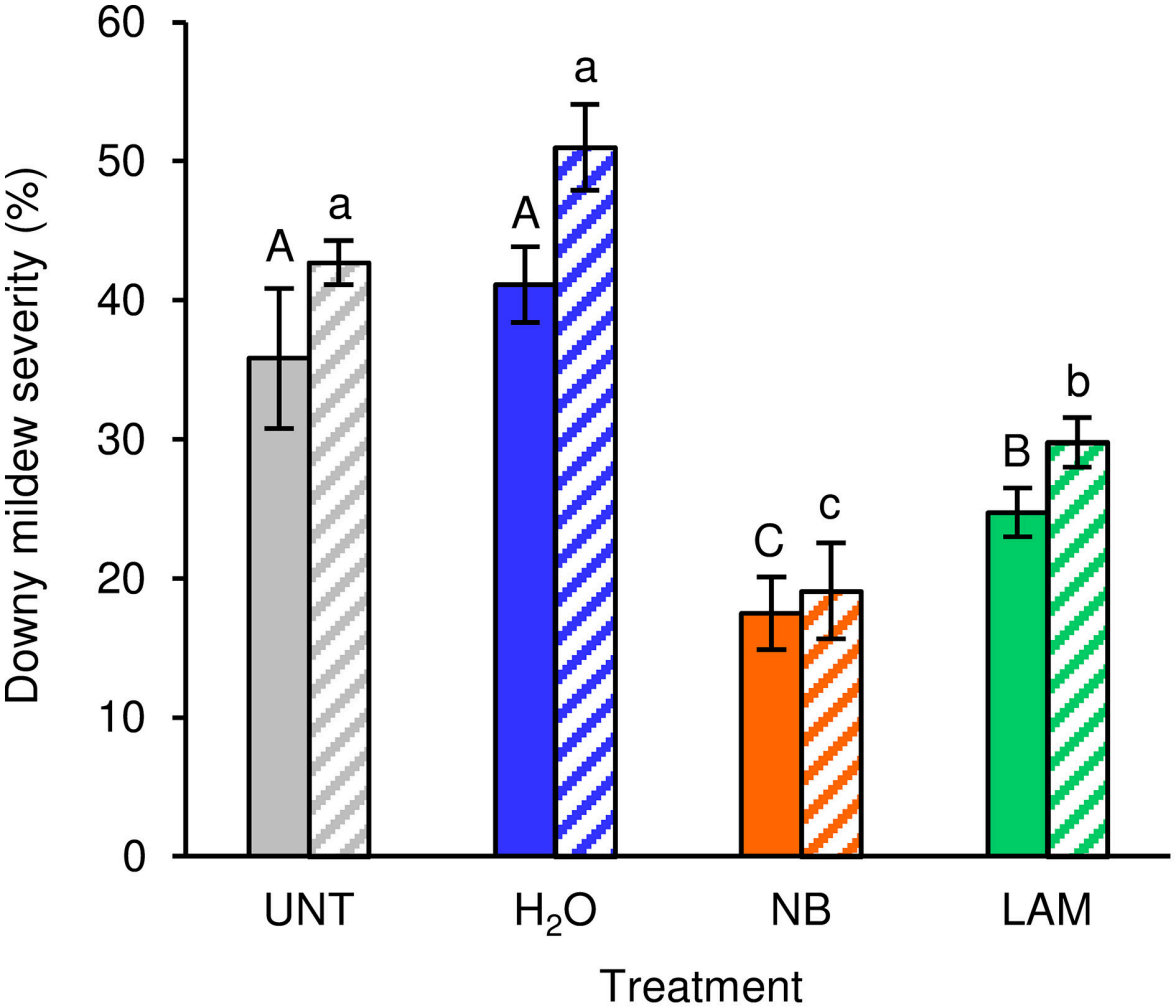
**Effect of leaf treatments on grapevine downy mildew severity under greenhouse conditions**. Plants were left untreated (UNT), or treated daily with water (H_2_O), nutrient broth (NB) or laminarin (LAM) three times before *Plasmopara viticola* inoculation. Disease severity was assessed as percentage of abaxial leaf area covered by *P. viticola* sporulation 7 days after inoculation. The *F*-test revealed differences between the experiments (*P* = 0.046) and each experiment was analyzed separately. Mean severity and standard error values of 12 replicates (plants) are presented for each treatment and experiment. Different uppercase and lowercase letters indicate significant differences among treatments according to Fisher's test (α = 0.05) in experiment 1 (solid bars) and experiment 2 (striped bars), respectively.

**Figure 3:**
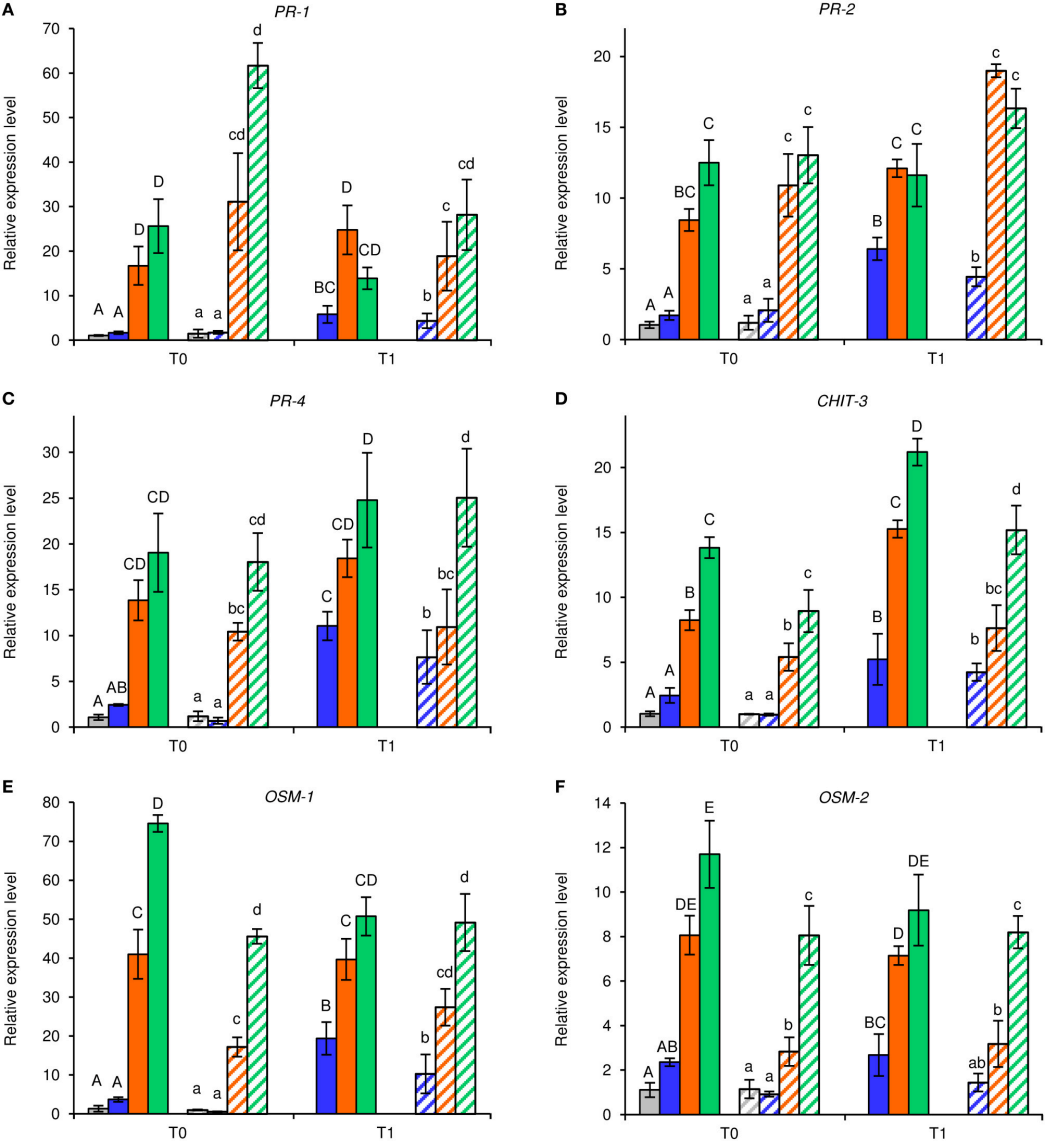
**Effect of leaf treatments on gene expression of grapevine plants under greenhouse conditions**. Grapevine plants were left untreated (gray), treated daily with water (blue), nutrient broth (orange) or laminarin (green) three times before *Plasmopara viticola* inoculation. Leaf samples were collected just before (T0) and 1 day after (T1) pathogen inoculation. Relative expression levels of genes encoding the pathogenesis-related (PR) protein 1 (*PR-1*; **A**), *PR-2* **(B)**, *PR-4* **(C)**, chitinase 3 (*CHIT-3*; **D**), osmotin 1 (*OSM-1*; **E**), and *OSM-2* **(F)** were assessed by quantitative real-time PCR. Relative expression levels were calculated using *Actin* as constitutive gene for normalization, and data were calibrated on untreated plants at T0. The *F*-test revealed differences between the experiments (*P*-values ranged from 0.0003 to 0.038 for the genes tested), and each experiment was analyzed separately for each gene. For each time point, mean levels and standard errors of relative expression are calculated based on three replicates (plants) for each treatment and experiment. For each gene, different uppercase and lowercase letters indicate significant differences among treatments and time points according to Fisher's test (α = 0.05) in experiment 1 (solid bars) and experiment 2 (striped bars), respectively.

Under greenhouse conditions, foliar applications of NB reduced downy mildew severity as compared with H_2_O-treated and UNT plants in the two different greenhouse experiments (Figure 2). Although a slight effect of the experiment was present (*F*-test, *P* = 0.046), the reduction of disease severity was greater in NB-treated plants (60.0 ± 1.3%) than in LAM-treated plants (34.6 ± 3.5%). Expression levels of the six previously mentioned defense-related genes (Table S1) were analyzed by qPCR in leaves collected at T0 and T1 of *P. viticola* inoculation (Figure 3). As expected, the expression levels of all tested genes were comparable in UNT and H_2_O-treated plants at T0, excluding the contribution of H_2_O treatment on defense gene modulation. The expression of all tested genes was induced by NB at T0 and it remained at a high level at T1; only the expression of *CHIT-3* was further enhanced at T1 in Exp 1. The expression levels of the defense genes *PR-1, PR-2, OSM-1* were higher in NB-treated plants with respect to H_2_O-treated plants at T0 and T1 (more than three- and two-fold, respectively), as well as those of *PR-4* at T0, *CHIT-3*, and *OSM*-2 at T0 and T1 of Exp 1. Conversely, *PR-4* in Exp 1 and Exp 2, *CHIT-3*, and *OSM-2* in Exp 2 showed comparable expression levels in NB and H_2_O-treated plants at T1. LAM treatment induced the expression of all tested genes at T0 and they remained at high expression levels at T1, with a further reinforcement of *CHIT-3* expression at T1 in both experiments. Expression levels of some genes were higher in LAM-treated plants in comparison to NB-treated plants, such as those of *CHIT-3, OSM-1* at T0, *OSM-2* in Exp 2, and *PR-4* at T1 in Exp 2. In agreement with previous findings (Perazzolli et al., 2011, 2012), *P. viticola* inoculation induced the expression of *PR-1, PR-2, PR-4, CHIT-3*, and *OSM-1* in H_2_O-treated plants at T1 in both experiments.

### Effects of grapevine treatments on the structure and composition of leaf microbial communities

Treatment with NB significantly increased the number of bacterial CFU per leaf unit as compared to controls (H_2_O-treated and UNT plants) at T0 and T1 in both experiments (File S1, Figures S1A,B). Conversely, H_2_O and LAM treatments did not affect bacterial CFU as compared to UNT plants. Considering the representative bacterial isolates originated from treated leaves, NB did not increase the percentage of bacteria with protease activity, siderophore production, or antagonistic activity against the oomycete *P. infestans* compared to H_2_O treatment (File S1, Table S3). Although the percentage of bacterial isolates with biocontrol activity against *P. viticola* did not increase after NB application, two isolates (*Pseudomonas* spp. and *Enterobacter* spp.) from NB-treated plants significantly reduced downy mildew severity on grapevine leaf disks (File S1, Figure S2). Culturable fungi were not affected by the treatments tested, except for the slight increase of fungal CFU in NB-treated plants of Exp 1 (Figures S1C,D).

The composition of bacterial and fungal communities was analyzed on leaves collected from UNT, H_2_O-, NB- and LAM-treated plants at T0 and T1 for the two independent greenhouse experiments. For bacterial (16S rRNA gene) and fungal (ITS) regions, 678,811 and 153,401 quality filtered reads were obtained, respectively (Tables S3–S5). Rarefaction curves (Figures S3, S4), Good's coverage and Chao1 indexes (Tables S4, S5) confirmed that the estimated microbial richness was sufficiently covered by the sequencing effort (File S1). OTU numbers, richness and microbial diversity estimated by the Simpson index highlighted differences of bacterial and fungal populations among experiments and grapevine treatments (File S1, Figures S5, S6).

**Figure 4:**
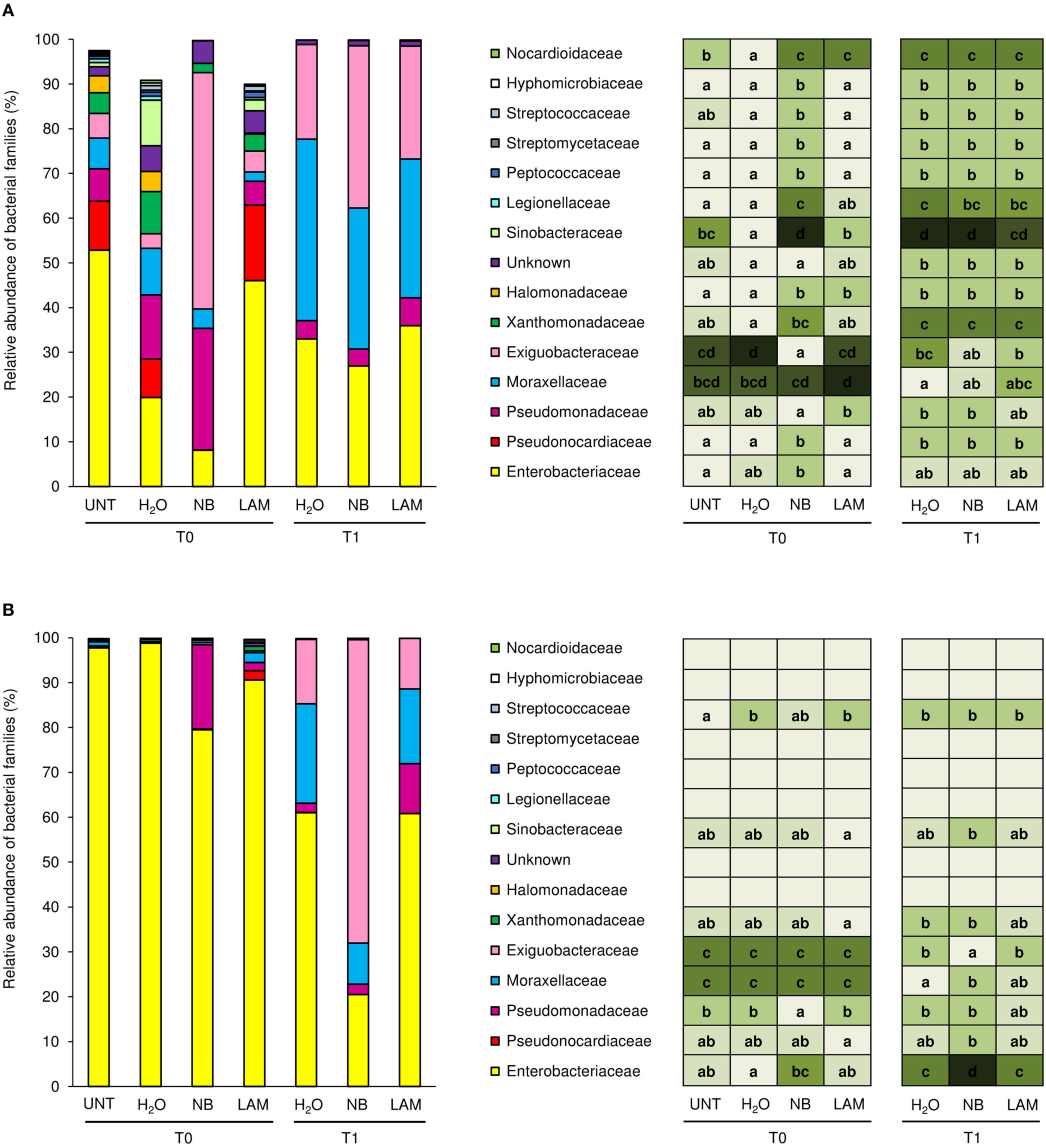
**Relative abundance of the dominant (more than 0.5% of relative abundance in at least one sample) bacterial families on grapevine leaves**. Percentages of relative abundance were determined for microbial populations of leaves of untreated plants (UNT), and plants treated with water (H_2_O), nutrient broth (NB), or laminarin (LAM) collected just before (T0) and 1 day after (T1) *Plasmopara viticola* inoculation in the Experiment 1 **(A)** and Experiment 2 **(B)**. Mean and standard error values of three replicates (each as a pool of two plants) were analyzed for each treatment and time point. For each taxon, the intensity of the color gradient and letters reported in the table indicate significant differences among treatments and time points according to Fisher's test (α = 0.05).

Almost the totality of bacterial reads (99.95 and 98.4%, respectively) were assigned to taxa at phylum (File S1, Figure S7, and Table S6) and family level (1404 OTU); 84 different bacterial families were identified in total, and the 15 dominant families were selected (more than 0.5% of relative abundance in at least one sample; Figure 4). Although all plants originated from the same nursery stock and were grown under the same controlled conditions, indigenous communities on UNT leaves differed between Exp 1 and Exp 2. In particular, the Enterobacteriaceae family comprised almost the totality of identified bacteria of UNT plants in Exp 2, while greater bacterial diversity was present on leaves of Exp 1. H_2_O treatment did not affect the proportions of bacterial families as compared with UNT plants in both experiments, except for Sinobacteraceae and Nocardioidaceae in Exp 1 (Figure 4A) and Streptococcaceae in Exp 2 (Figure 4B), and H_2_O-treated plants were used as reference control for treatment comparisons. In Exp 1, the abundance of Exiguobacteraceae significantly increased on NB-treated plants in comparison to H_2_O-treated plants on leaves collected at T0, while the proportions of Pseudonocardiaceae, Xanthomonadaceae, Halomonadvaceae, Sinobacteraceae, Legionellaceae, Peptococcaceae, Streptomycetaceae, Streptococcaceae, Hyphomicrobiaceae, and Nocardioidaceae decreased (Figure 4A). Furthermore, lower abundance of Halomonadaceae, Sinobacteraceae, and Nocardioidaceae was observed on LAM-treated plants in comparison to H_2_O-treated plants. At T1, relative abundances of bacterial families were comparable on H_2_O-, NB-, and LAM-treated plants. For T0 samples of Exp 2, the abundance of Enterobacteriaceae was lower on NB-treated plants with respect to H_2_O-treated plants, while that of Pseudomonadaceae was greater (Figure 4B). The presence of Exiguobacteraceae increased on NB-treated plants as compared to H_2_O-treated plants at T1, while proportions of Enterobacteriaceae and Moraxellaceae decreased. The relative abundance of all the dominant families was comparable on LAM- and H_2_O-treated plants at both time points.

**Figure 5:**
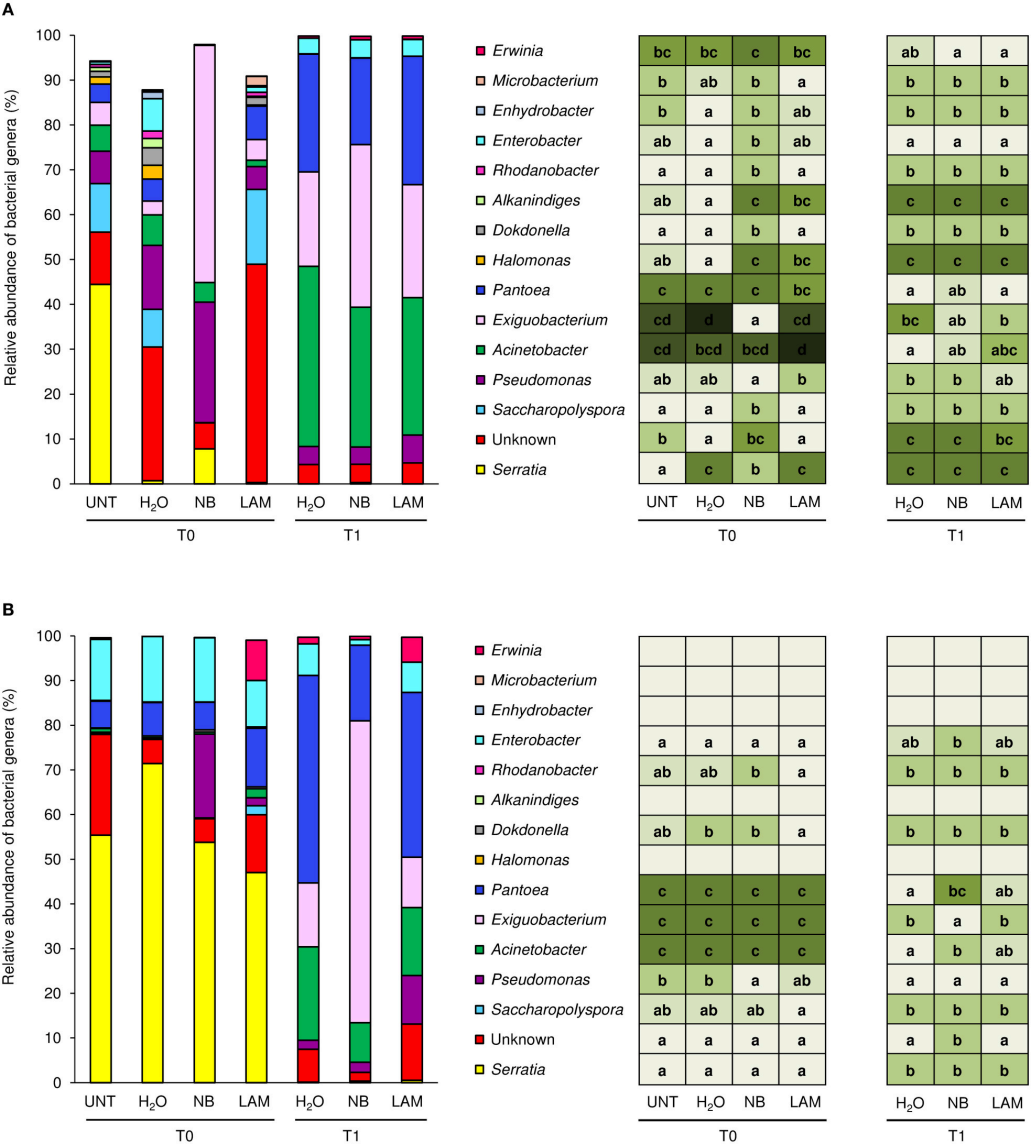
**Relative abundance of the dominant (more than 2% of relative abundance in at least one sample) bacterial genera on grapevine leaves**. Percentages of relative abundance were determined for leaves of untreated plants (UNT), and plants treated with water (H_2_O), nutrient broth (NB), or laminarin (LAM) collected just before (T0) and 1 day after (T1) *Plasmopara viticola* inoculation in the experiment 1 **(A)** and experiment 2 **(B)**. Mean and standard error values of three replicates (each as a pool of two plants) were analyzed for each treatment and time point. For each taxon, the intensity of the color gradient and letters reported in the table indicate significant differences among treatments and time points according to Fisher's test (α = 0.05).

Of bacterial reads, 87.3% were assigned to taxa at the genus level (885 OTU), 150 and 70 different bacterial genera and species were identified, respectively. Relative abundances of the dominant genera (Figure 5) and dominant species (Figure S8) differed by treatment, time point and experiment. H_2_O treatment did not modify genera proportions as compared with UNT plants, only in Exp 1 the genus *Serratia* decreased, while the Unknown and *Enhydrobacter* genera increased (Figure 5A). On leaves collected at T0, levels of the *Serratia* and *Exiguobacterium* genera significantly increased on NB-treated plants in comparison to H_2_O-treated plants in Exp 1, whereas those of Unknown, *Saccharopolyspora, Halomonas, Dokdonella, Alkanindiges, Rhodanobacter, Enterobacter*, and *Enhydrobacter* decreased (Figure 5A). Furthermore, lower abundances of *Halomonas* and *Alkanindiges* were observed on LAM-treated plants in comparison to H_2_O-treated plants. At T1, relative abundances of bacterial genera were comparable on H_2_O-, NB-, and LAM-treated plants. In Exp 2, the abundance of *Pseudomonas* was increased by NB with respect to H_2_O-treated plants (Figure 5B). At T1, the presence of *Exiguobacterium* increased on NB-treated plants in comparison to H_2_O-treated plants, while the proportions of Unknown, *Acinetobacter* and *Pantoea* were reduced. Relative abundances of all dominant genera were comparable on LAM- and H_2_O-treated plants at both time points, except for an increase in *Dokdonella* proportions at T0. Although abundances of genera related to the biocontrol of downy mildew were scarcely affected by grapevine treatments, it is remarkable that in Exp 2 the abundance of *Lysobacter* was greater on NB-treated plants in comparison to H_2_O-treated plants at T0 (Table 1).

**Table 1:** **Relative abundance of bacterial and fungal genera in the phyllosphere microbial populations of experiment 1 and experiment 2, which comprise known biocontrol agents against *Plasmopara viticola***.

**Genus^1^**	**Relative abundance^2^**						
	**UNT T0**	**H_2_O T0**	**NB T0**	**LAM T0**	**H_2_O T1**	**NB T1**	**LAM T1**
**EXPERIMENT 1**							
*Bacillus*	0.00 ± 0^a^	0.02 ± 0.02^a^	0.00 ± 0^a^	0.00 ± 0^a^	0.00 ± 0^a^	0.00 ± 0^a^	0.00 ± 0^a^
*Lysobacter*	0.64 ± 0.24^a^	0.78 ± 0.29^a^	0.57 ± 0.46^a^	0.19 ± 0.12^ab^	0.00 ± 0^b^	0.00 ± 0^b^	0.00 ± 0^b^
*Stenotrophomonas*	0.99 ± 0.85^ab^	0.16 ± 0.02^abc^	1.41 ± 1.04^a^	0.16 ± 0.10^abc^	0.00 ± 0^c^	0.02 ± 0.02^bc^	0.00 ± 0^c^
*Alternaria*	0.00 ± 0^c^	0.02 ± 0.02^bc^	0.00 ± 0^c^	0.00 ± 0^c^	0.00 ± 0^c^	0.10 ± 0.05^a^	0.03 ± 0.02^b^
*Aureobasidium*	0.00 ± 0^b^	0.05 ± 0.03^a^	0.02 ± 0.02^ab^	0.00 ± 0^b^	0.00 ± 0^b^	0.00 ± 0^b^	0.05 ± 0.03^a^
*Fusarium*	0.00 ± 0^a^	0.00 ± 0^a^	0.00 ± 0^a^	0.00 ± 0^a^	0.00 ± 0^a^	0.00 ± 0^a^	0.03 ± 0.03^a^
*Trichoderma*	0.00 ± 0^a^	0.00 ± 0^a^	0.00 ± 0^a^	0.00 ± 0^a^	0.00 ± 0^a^	0.02 ± 0.02^a^	0.00 ± 0^a^
*Penicillium*	4.95 ± 1.00^a^	1.48 ± 0.18^b^	2.21 ± 0.79^ab^	2.27 ± 0.99^ab^	2.68 ± 0.90^ab^	2.34 ± 0.70^ab^	2.37 ± 0.94^ab^
*Acremonium*	2.47 ± 0.93^a^	0.98 ± 0.16^a^	0.98 ± 0.36^a^	4.38 ± 2.96^a^	1.77 ± 1.16^a^	1.35 ± 070^a^	2.95 ± 0.49^a^
**EXPERIMENT 2**							
*Bacillus*	0.00 ± 0^a^	0.00 ± 0^a^	0.00 ± 0^a^	0.00 ± 0^a^	0.00 ± 0^a^	0.00 ± 0^a^	0.00 ± 0^a^
*Lysobacter*	0.02 ± 0.02^ab^	0.00 ± 0^b^	0.16 ± 0.13^a^	0.07 ± 0.07^ab^	0.00 ± 0^b^	0.00 ± 0^b^	0.00 ± 0^b^
*Stenotrophomonas*	0.00 ± 0^a^	0.00 ± 0^a^	0.00 ± 0^a^	0.00 ± 0^a^	0.00 ± 0^a^	0.00 ± 0^a^	0.00 ± 0^a^
*Alternaria*	0.00 ± 0^b^	0.00 ± 0^b^	0.00 ± 0^b^	0.07 ± 0.03^a^	0.00 ± 0^b^	0.07 ± 0.07^ab^	0.00 ± 0^b^
*Aureobasidium*	0.02 ± 0.02^b^	0.00 ± 0^b^	0.02 ± 0.02^b^	0.00 ± 0^b^	0.10 ± 0.03^ab^	0.56 ± 0.31^a^	0.34 ± 0.22^a^
*Fusarium*	0.03 ± 0.03^a^	0.00 ± 0^a^	0.00 ± 0^a^	0.12 ± 0.12^a^	0.00 ± 0^a^	0.00 ± 0^a^	0.00 ± 0^a^
*Trichoderma*	0.02 ± 0.02^b^	0.00 ± 0^b^	0.05 ± 0.05^ab^	0.02 ± 0.02^b^	0.00 ± 0^b^	0.08 ± 0.02^a^	0.00 ± 0^b^
*Penicillium*	17.15 ± 4.62^a^	5.44 ± 0.50^a^	12.35 ± 8.27^a^	19.44 ± 10.79^a^	6.79 ± 1.08^a^	5.22 ± 1.35^a^	4.21 ± 1.17^a^
*Acremonium*	11.49 ± 8.34^a^	1.55 ± 0.59^a^	10.21 ± 9.88^a^	4.29 ± 2.02^a^	9.43 ± 3.80^a^	2.71 ± 1.73^a^	14.40 ± 13.47^a^

1 *Bacterial genera comprising known biocontrol agents against downy mildew: Bacillus spp., Stenotrophomonas maltophilia (Gessler et al., 2011), Lysobacter capsici (Puopolo et al., 2014), Alternaria alternata (Musetti et al., 2006), Aureobasidium pullulans (Harm et al., 2011), Fusarium proliferatum (Falk et al., 1996), Trichoderma harzianum (Perazzolli et al., 2011), Penicillium chrysogenum (Thuerig et al., 2006), and Acremonium byssoides (Burruano et al., 2008) identified on grapevine leaves in the two experiments under greenhouse conditions (Experiment 1 and Experiment 2)*.

2 *Percentages of relative abundance of bacterial and fungal genera with possible biocontrol activities were determined for leaves of untreated plants (UNT), plants treated with water (H_2_O), nutrient broth (NB) or laminarin (LAM), collected just before (T0) and 1 day after (T1) Plasmopara viticola inoculation and normalized to the lowest number of quality filtered reads in the Experiment 1 and Experiment 2. The mean and standard error values of three replicates (each as a pool of two plants) were analyzed for each treatment and each time point. For each genus, different superscript letters indicate the significant differences according to Fisher's test (α = 0.05)*.

Concerning fungal populations, 34, 53, and 87 different families, genera and species were identified in total, respectively (Table S7). Proportions of the ten dominant families (Figure S9), seven dominant genera (Figure S10) and 15 dominant species (Figure S11) were homogeneous between the two experiments and only slightly affected by treatments and time points (File S1). As regard to fungal genera related to the biocontrol of downy mildew, in Exp 1 greater abundance of *Alternaria* spp. was detected on NB-treated plants in comparison to H_2_O-treated plants at T1, while in Exp 2 the relative abundance of *Trichoderma* spp. was greater on NB-treated plants in comparison to H_2_O-treated plants at T1 (Table 1).

**Figure 6:**
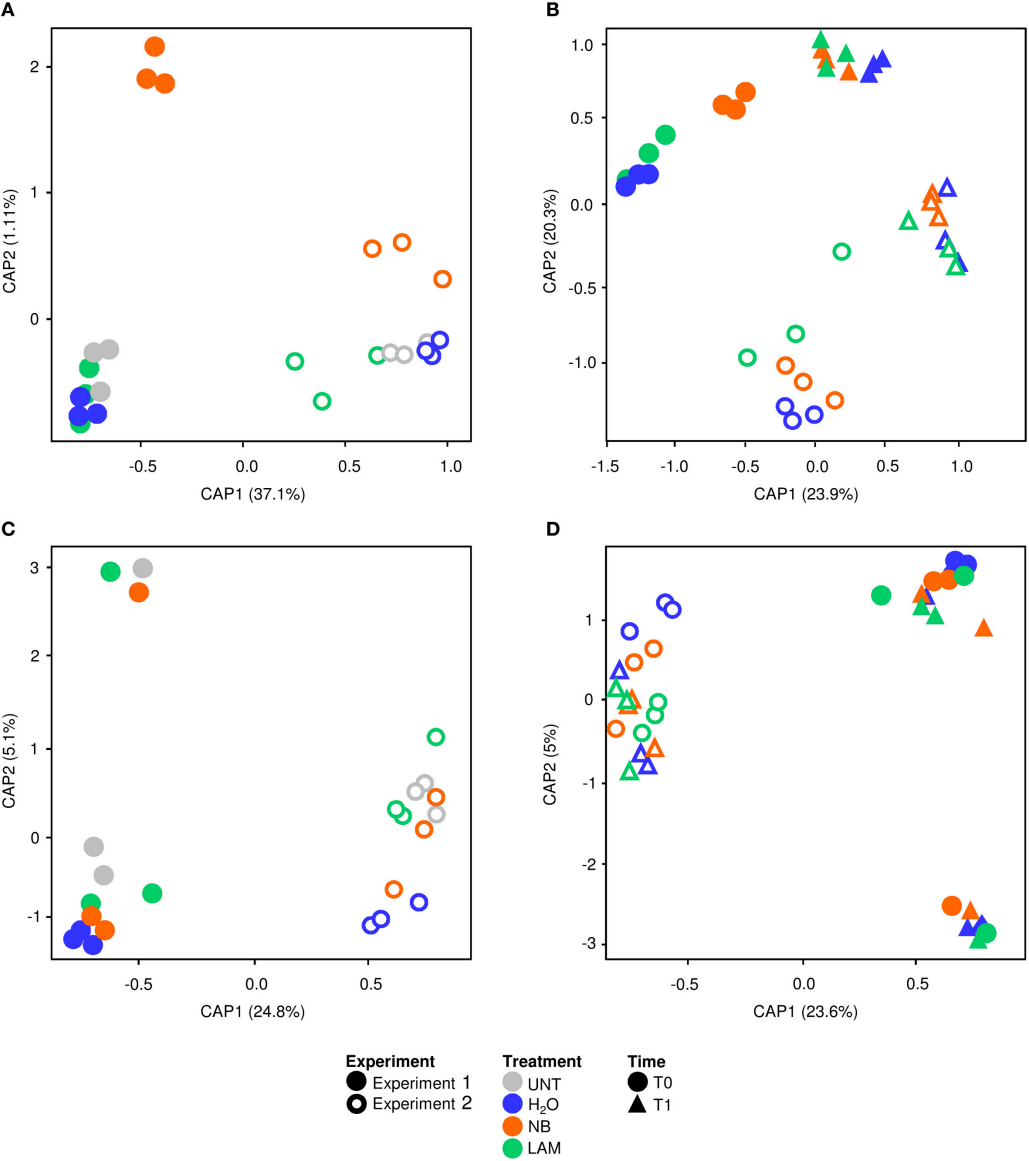
**Canonical analysis of principal coordinates (CAP) of leaf microbial communities**. CAP of bacterial **(A,B)** and fungal **(C,D)** communities were obtained with the vegan package using Bray-Curtis dissimilarity matrix on data of untreated plants (UNT), and plants treated with water (H_2_O), nutrient broth (NB), or laminarin (LAM) collected just before (T0) and 1 day after (T1) *Plasmopara viticola* inoculation. CAP was carried out for T0 samples **(A,C)**, and for T0 and T1 samples excluding UNT **(B,D)**. Results of PERMANOVA and Permutation tests obtained with ADONIS function and a Bray-Curtis dissimilarity matrix on bacterial and fungal data are reported in Tables S8, S9, respectively.

Global effects of experiments, treatments and time points on bacterial and fungal diversity were examined using PERMANOVA and CAP analyses. PERMANOVA of bacterial samples collected at T0 indicated significant differences (*P* = 0.0001) among experiments and treatments (Table S8). CAP validated these results, and the first principal coordinate discriminated samples of Exp 1 from those of Exp 2 at T0, while the second axis highlighted differences among treatments (Figure 6A), with significant differences among experiments (*P* = 0.001) and treatments (*P* = 0.002) according to permutation tests on CAP (Table S8). Permutation pairwise comparisons showed significant differences between NB-treated and UNT plants (*P* = 0.0044), between NB- and H_2_O-treated plants (*P* = 0.0062), but not between LAM- and H_2_O-treated (*P* = 0.0788) and LAM-treated and UNT (*P* = 0.1536) plants (Table S8). Considering samples collected at T0 and T1, PERMANOVA identified significant differences among treatments (*P* = 0.0077), time points (*P* = 0.0001) and experiments (*P* = 0.0001; Table S8). CAP discriminated the two time points and the two experiments on the first and the second axis, respectively (Figure 6B), and permutation tests on CAP supported significant differences of bacterial communities among time points (*P* = 0.001), experiments (*P* = 0.001) and treatments (*P* = 0.011). Permutation pairwise comparisons obtained for samples collected at T1 revealed no significant effects of grapevine treatments (Table S8), and indicated that effects on bacterial populations occurred at T0. The CAP of fungal data discriminated the two experiments on the first axis considering samples collected at T0 (Figure 6C) or at T0 and T1 (Figure 6D), and permutation tests confirmed significant differences between experiments (*P* = 0.001; Table S9). PERMANOVA detected no significant difference among treatments and time points, in agreement with permutation test results applied to CAP (Table S9).

## Discussion

Several alternatives have been proposed to reduce the massive use of chemical pesticides in viticulture (Gessler et al., 2011; Delaunois et al., 2014) and the induction of plant resistance through the use of protein-based elicitors seems to be a promising additional tool (Lachhab et al., 2014, 2016; Nesler et al., 2015). In particular, the protein derivative named NB does not raise toxicological or ecotoxicological concerns, and it could represent a valid control product for integrated plant protection programs (Nesler et al., 2015). However, beyond its properties as a resistance inducer, NB could affect the composition of phyllosphere microbial populations, which in turn might contribute to resistance induction and/or display direct biocontrol properties.

Under axenic conditions, NB strongly reduced downy mildew symptoms and induced the expression of five defense-related genes (*PR-2, PR-4, OSM-1, OSM-2*, and *CHIT-3*), suggesting that it was effective against *P. viticola* through the induction of grapevine resistance. The expression of these genes remained at a high level even after pathogen inoculation (at T1), indicating that grapevine resistance induced by NB plays a major role in limiting host colonization during the early stages of *P. viticola* infection. Likewise, the relevance of a rapid up-regulation of defense genes, i.e. within a few hours after inoculation, has been demonstrated for the response against downy mildew in resistant genotypes (Polesani et al., 2010; Casagrande et al., 2011). Our data indicate that the preventive foliar treatment with NB reduced downy mildew symptoms under greenhouse conditions, through the induction of all defense-related genes tested, including *PR-1*. Moreover, the expression of three genes was further enhanced in NB-treated plants in response to *P. viticola* inoculation, such as *PR-4* and *OSM-1* under axenic conditions, and *CHIT-3* in Exp 1 under greenhouse conditions. Thus, marker genes of SA and JA pathways, such as *PR-1* and *PR-4*, respectively (Hamiduzzaman et al., 2005), were induced by NB under greenhouse conditions, suggesting the activation of both signaling pathways (Nesler et al., 2015). Expression profiles of *in vitro*-grown plants partially differed from those of greenhouse-grown plants, and the SA marker (*PR-1*) was not induced by NB under axenic conditions. Although different expression profiles between axenic and greenhouse conditions could be related to different growing conditions of the plants, they could also be associated with changes induced by NB in the phyllosphere microbiota of greenhouse-grown plants. Some components of protein-derived products can be metabolized by the phyllosphere microorganisms, thus modifying the properties and efficacy of the originally applied product (Colla et al., 2015). In particular, NB might stimulate the JA-mediated pathways under axenic conditions, and the phyllosphere microorganisms could contribute to the activation of SA pathways under greenhouse conditions. Therefore, changes in phyllosphere microbiota composition could modify the plant signals stimulated by the protein-based resistance inducer. This second scenario, coupled with the fact that plants are naturally exposed to a wide variety of microorganisms under natural conditions and that plant resistance might be already partially activated, could explain the greater efficacy of NB under axenic conditions with respect to greenhouse conditions. Although LAM induced the expression of all defense-related genes tested, with a higher expression of some of them in comparison with NB treatment (*CHIT-3, OSM-1, OSM-2*, and *PR-4*), it showed lower efficacy than NB against downy mildew. These results suggest that multiple mechanisms of action are involved in the biocontrol activity of NB and that additional biotic factors, i.e. the phyllosphere microbiota could influence the efficacy against downy mildew.

Pyrosequencing analysis allowed the dissection of compositions and modifications of the microbial populations residing on the grapevine phyllosphere after the treatments tested. Even though plants in the study originated from the same nursery stock, and were grown under the same controlled conditions, significant differences among bacterial populations were found between the two greenhouse experiments. The plant phyllosphere act as an open system, and the structure of its microbial community reflects immigration, survival and growth of microbial colonists, which in turn is influenced by numerous environmental factors, in addition to leaf physico-chemical properties (Whipps et al., 2008a). The high variability among microbial populations residing on grapevine leaves in time and space is in agreement to what already observed in field experiments (Perazzolli et al., 2014). Subsequently, changes occurred in leaf bacterial and fungal populations after the treatments tested were affected by the composition of the originally residing microbiota. The dominant phyla were Proteobacteria and Actinobacteria, as already reported for grapevine phyllosphere (Leveau and Tech, 2011; Perazzolli et al., 2014; Pinto et al., 2014) and rhizosphere microorganisms (Zarraonaindia et al., 2015). It has been shown that microbial communities associated with grapevine leaves share a great proportion of taxa with soil populations, suggesting that the soil is the main microbial reservoir of the aboveground communities (Zarraonaindia et al., 2015). *Xanthomonadales, Rhizobiales*, and *Actinomycetales* were shown to be the dominant bacterial orders of grapevine root and rhizosphere communities (Zarraonaindia et al., 2015), and Xanthomonadaceae, Hyphomicrobiaceae, and Pseudonocardiaceae were among the most abundant bacterial families in our samples, respectively. Particularly, *Pseudomonas, Acinetobacter, Exiguobacterium, Pantoea, Alkanindiges, Enterobacter*, and *Erwinia* were among the genera with highest presence, as previously reported for grapevine leaves (Bulgari et al., 2009; Leveau and Tech, 2011; Martins et al., 2013; Perazzolli et al., 2014; Pinto et al., 2014). Bacterial community structure was globally affected by time points and by NB treatment in both experiments, while no effect was seen from LAM treatment. NB application possibly act as nutritional substrate for some bacteria and increased abundances of the *Exiguobacterium* genus as compared with H_2_O treatment at T0 in Exp 1 and T1 in Exp 2. Interactions between *Exiguobacterium acetylicum* and two other bacteria (namely *Microbacterium* spp. and *Pantoea agglomerans*) have been reported to contribute to the suppression of the wheat root disease caused by *Rhizoctonia solani* (Barnett et al., 2006), indicating potential biocontrol properties of some *Exiguobacterium* strains. In Exp 2, the NB treatment increased the proportion of the Pseudomonadaceae family and the *Pseudomonas* genus as compared with UNT plants and H_2_O-treated plants at T0, and *P. viridiflava* and *P. veronii* levels showed the same trend. Some species of this genera are known as active resistance inducers (Van Wees et al., 2008) and biocontrol agents, for their ability to produce proteases (Elad, 2000), siderophores (Van Wees et al., 2008) and antimicrobial metabolites (Ligon et al., 2000). Specifically for grapevine plants, members of *Pseudomonas* have been demonstrated to effectively control *Botrytis cinerea* infections by inducing resistance mechanisms (Trotel-Aziz et al., 2008). Finally, the NB treatment increased also the proportion of *P. alcaligenes* at T0 in Exp 1, and this species has been reported as biocontrol agent against *Fusarium oxysporum* (Akhtar et al., 2010). In Exp 2, the Enterobacteriaceae family accounted for the majority of bacterial OTU at T0, and its abundance was affected by NB at both time points. One of the dominant species was *Serratia marcescens*, which significantly increased by NB as compared with respect to H_2_O-treated plants at T0 in Exp 1*. S. marcescens* was reported as biocontrol agent against the soil-borne fungus *Magnaporthe poae* (Kobayashi et al., 1995) and the rice pathogen *Magnaporthe oryzae* (Jaiganesh et al., 2007). A strain of *Lysobacter capsici* reduced downy mildew symptoms in grapevine (Puopolo et al., 2014), and the abundance of the *Lysobacter* genus on grapevine leaves increased as a result of NB treatment at T0 in Exp 2. Grapevine bacterial pathogens, such as *Agrobacterium vitis, Xylella fastidiosa*, and *Xylophilus ampelinus* (Armijo et al., 2016) were not detected in the samples analyzed, and further studies are required to better characterize possible side effects of NB on bacterial phytopathogens. However, negligible effects on *X. fastidiosa* are highly possible, due to its transmission to new host plants exclusively by insect vectors (Armijo et al., 2016). Although more sensitive analyses are required to precisely quantify human pathogenic strains, *Salmonella* spp., *Legionella* spp., and *Escherichia* spp. were underrepresented on grapevine leaves and their abundances were not affected by the NB treatment, suggesting a minimal risk in term of increase of microorganisms potentially dangerous for human health.

The structure of fungal communities were similar in Exp 1 and Exp 2 and they were not globally affected by NB and LAM treatment. The fungal microbiota of grapevine leaves was strongly dominated by the Ascomycota phylum, as reported for other plants (Jumpponen and Jones, 2009), and by the Trichocomaceae family. A substantial part of sequenced reads was attributed to the Unknown group, which probably represented environmental sequences of unculturable fungi. The most common genera identified on grapevine leaves were *Aspergillus, Penicillium*, and *Acremonium*, as reported for other plants (Inacio et al., 2002; Whipps et al., 2008b). The NB treatment modified abundances of some specific fungal taxa, such as the *Alternaria* genus at T1 in Exp 1, and *A. alternata* was able to control *P. viticola* on leaf disks (Musetti et al., 2006). Relative abundances of *Trichoderma* spp. and *Aureobasidium* spp. were increased by the NB treatment at T1 in Exp 2. A strain of *Trichoderma harzianum* induces grapevine resistance (Perazzolli et al., 2011), and an isolate of *Aureobasidium pullulans* partially protects against downy mildew (Harm et al., 2011). Summarizing, the preventive foliar application of NB on grapevine partially alters the structures and dynamics of bacterial populations, and specific differences highlighted effects on some genera that may be related to biocontrol activity and resistance induction. On the other hand, the fungal communities on grapevine leaves were more stable than bacterial populations in the time-frame studied. This may be related to shorter generation time of the bacteria and/or the preference of bacteria for protein and amino acids as nutritional source (Vorholt, 2012). Another possible reason for stability may be the longer generation time of fungi that did not afford appreciable modifications within the short time of the experiment (4 days).

Although culturable microorganisms represent a limited fraction of the community, they are the most likely to be influenced by NB, which is a laboratory microbiological medium. The increase of culturable microorganisms on NB-treated plants confirmed that the protein derivative had a nutritional role that affected mainly bacteria. However, *in vitro* assays highlighted that the NB treatment did not affect proportions of bacterial isolates with proteolytic activity, siderophore production and antagonistic activity against *P. infestans*, suggesting the absence of positive selection of potential biocontrol agents against oomycetes. Although proportions of biocontrol strains effective against *P. viticola* were not increased by the NB treatment, two isolates from NB-treated plants showed biocontrol activity against *P. viticola* on leaf disks. In short, NB leaf application on greenhouse-grown grapevines increased the number of culturable bacteria and slightly altered the structure of the residing phyllosphere microbiota. These changes may contribute to pathogen control resulting from competition for space or from other biocontrol strategies, resistance induction included. Thus, functional properties of the phyllosphere microbiota against plant diseases (Vorholt, 2012; Ritpitakphong et al., 2016) could be improved by application of nutritional substrates for leaf microorganisms.

In conclusion, NB could represent a promising alternative for the control of *P. viticola* on grapevines, considering its natural origin and the multiple mechanisms of action. The application of a protein-based resistance inducer to prevent grapevine diseases could bring appreciable advantages, such as the absence of toxicity for the environment and the activation of defense mechanisms that protects plants against different diseases, such as powdery (Nesler et al., 2015) and downy mildew. Moreover, weekly applications of NB did not produce any negative effect on grapevine growth and yield in two different seasons, indicating minimal risks for grape production and quality (Nesler et al., 2015). As demonstrated for the control of powdery mildew (Nesler et al., 2015), the reduction of downy mildew symptoms is mainly based on the induction of defense mechanisms in grapevine, involving multiple signaling pathways. Furthermore, NB increased the number of culturable phyllosphere microorganisms and changed proportions of some taxa that have previously been linked to the biological control of plant pathogens. Thus, modifications of the phyllosphere microbiota due to NB treatment may provide a partial contribution to the control of downy mildew. Although, changes in the microbial populations depend on the indigenous communities originally residing on grapevine leaves before treatment, levels of disease reduction and defense gene modulation of NB-treated plants were consistent among the experiments. The plant phyllosphere act as an open system and population dynamics are more complex than expected, suggesting that the resolution power of current meta-barcoding approaches of metagenomics is still insufficient to link modifications in microbial composition to functional changes. Further functional analyses of NB-affected populations, for example with metatranscriptomic approaches, are required to precisely characterize their effective contribute in term of disease control.

## Author contributions

MC analyzed the sequencing data and wrote the paper MPe designed the experiments, analyzed the data and wrote the paper LA carried out the bioinformatics and statistical analysis of the sequencing data AN carried out the experiments under greenhouse condition, the gene expression analysis and the characterization of culturable microorganisms ET carried out the gene expression analysis and the characterization of culturable microorganisms. PLB carried out the experiments with *in vitro*-grown plants MPi carried out the ITS and 16S sequencing GP carried out the experiments under greenhouse conditions and the characterization of culturable microorganisms IP conceived the study, designed the experiment, coordinated all research activities and wrote the paper. All authors revised and approved the final manuscript.

### Conflict of interest statement

The authors declare that the research was conducted in the absence of any commercial or financial relationships that could be construed as a potential conflict of interest.

## Abbreviations

CAP: canonical analysis of principal coordinates
CFU: colony forming units
CHIT-3: chitinase 3
Exp1: experiment 1
Exp2: Experiment 2
H_2_O: water
ITS: internal transcribed spacer
JA: jasmonic acid
LAM: commercial laminarin-based product
NB: nutrient broth
OSM-1: osmotin 1
OTU: operational taxonomic units
PERMANOVA: permutational multivariate analysis of variance
PR: pathogenesis related
PR-1: pathogenesis related protein 1
PR-2: pathogenesis related protein 2
PR-4: pathogenesis related protein 4
qPCR: quantitative real-time PCR
SA: salicylic acid
T0: just before *Plasmopara viticola* inoculation
T1: 1 day after *Plasmopara viticola* inoculation
UNT: untreated.
