# Supplementary material for: Leaf Treatments with a Protein-Based Resistance Inducer Partially Modify Phyllosphere Microbial Communities of Grapevine
Source: Front Plant Sci. 2016 Jul 19;7:1053. doi: 10.3389/fpls.2016.01053 (PMC4949236; doi:10.3389/fpls.2016.01053)
Supplement: Supplementary file 10 [file Presentation1.PDF]

Characterization of Culturable Leaf Microorganisms

In order to evaluate the effect of NB on phyllosphere microorganisms, viability of culturable bacteria and fungi on grapevine leaves was assessed by a plating method on selective media. Numbers of colony forming units (CFU) of culturable bacteria were greater on NB-treated plants in comparison to H_2O-treated and UNT plants at T0 and T1 in both experiments (Figures S1A and S1B). CFU numbers were generally greater at T1 in comparison to T0 on H_2O-treated plants, and the absence of bacteria in the inoculum suspension (data not shown) indicated a possible stimulation by the moist chamber used for pathogen inoculation. CFU of culturable fungi were not significantly influenced by the treatments tested, except for an increment caused by NB on grapevines of Exp 1 (Figures S1C and S1D).

Because NB application increased the number of culturable bacteria on grapevine leaves, some representative bacterial isolates (60 from the Exp 1, and 37 from the Exp 2) were selected visually based on colony morphological features from samples of H_2O- and NB-treated plants. Indicators of biological control properties were analyzed for each representative isolate: percentages of bacteria with protease activity, siderophore production and antagonistic activities against the oomycete *Phytophthora infestans* were comparable among isolates collected from NB- and H_2O-treated plants at both time points (Table S2). Furthermore, seven and six bacterial isolates were randomly selected for Exp 1 and Exp 2, respectively, and tested against *Plasmopara viticola* on leaf discs. Two bacterial isolates collected from NB-treated plants significantly reduce downy mildew severity on leaf disks with a disease reduction lower than that of the biocontrol agent *Lysobacter capsici* AZ78 (Figure S2). These isolates corresponded to a *Pseudomonas* spp. (KU596386; T1_NB_7 of Exp 1) and an *Enterobacter* spp. (KU596387; T1_NB_13 of Exp 2) by sequencing of the V6-V8 hypervariable region of the 16S rRNA gene.

Identification, Richness and Diversity of Leaf Microbial Communities

Pyrosequencing analysis of bacterial (16S rRNA gene) and fungal (ITS fragment) amplicons (Table S3) was carried out to identify microorganisms collected from grapevine leaves of plants under greenhouse conditions. After filtering out low-quality reads and short sequences, 403,900 (Exp 1) and 274,911 (Exp 2) reads of bacteria, and 78,542 (Exp 1) and 74,859 (Exp 2) reads of fungi were obtained (Tables S4 and S5). The total number of filtered reads for each replicate ranged from 2,703 to 52,719 (Exp 1) and from 1,415 to 54,709 (Exp 2) for bacteria (Table S4), and from 1,979 to 8,652 (Exp 1) and from 1,982 to 10,174 (Exp 2) for fungi (Table S5). Good’s coverage was used to estimate the completeness of sampling with a probability calculation based on randomly selected sequences, and it ranged from 82.1 to 97.5% (Exp 1) and from 95.8 to 99.3% (Exp 2) for bacterial data (Table S4), and from 95.8 to 98.0% (Exp 1) and from 95.6 to 98.1% (Exp 2) for fungal data (Table S5). Likewise, rarefaction curves confirmed that a sufficient saturation was reached for both bacterial and fungal populations of each replicate (Figures S3 and S4). Chao1 index revealed that more than 88% and 74% for bacteria (Table S4) and more than 79% and 67% for fungi (Table S5) of the estimated richness was covered by the sequencing effort in Exp 1 and Exp 2, respectively.

Bacterial and fungal data were normalized to the lowest number of filtered reads (1415 in H_2O at T1 replicate A in Exp 2, and 1979 in H_2O at T1 replicate C of Exp1, respectively) and OTU were recalculated for each sample. In Exp 1, richness of bacterial communities was lower on NB-treated plants in comparison to H_2O-treated and LAM-treated plants at T0, while OTU numbers at T1 were comparable among all treatments (Figure S5A). In Exp 2, bacterial richness was significantly lower on H_2O-treated plants at T0 with respect to all other treatments at T1. Richness of fungal populations was comparable among treatments in Exp 2, and it was greater on H_2O-treated plants at T0 in comparison to T1 (Figure S5B).

Bacterial diversity estimated by the Simpson index significantly increased by the grapevine treatments at both time points compared to UNT plants in Exp 1, except for NB-treated plants at T0 and LAM-treated plants at T1. Conversely, in Exp 2 the diversity estimator had the highest value on NB-treated leaves at T1 and the lowest value on H_2O-treated leaves at T0 (Figure S6A). For fungal populations, the Simpson index significantly differed only between T0 and T1 of H_2O-treated plants in Exp 1, and between H_2O-treated plants at T0 and LAM-treated plants at T1 in Exp 2 (Figure S6B).

Distribution of Bacterial Phyla among Experiments, Treatments and Time Points

Almost the totality of bacterial reads (99.95%) were assigned to taxa at phylum level, 11 different bacterial phyla were detected in total (Table S6) and 7 dominant phyla were presented (more than 0.4 % of relative abundance in at least one sample, Figure S7). In Exp 1, the relative abundance of Cyanobacteria, Unknown phyla and Planctomycetes was greater on H_2O-treated plants in comparison to UNT plants (Figure S7A). On leaves collected at T0, the abundance of Firmicutes was greater on NB-treated plants in comparison to H_2O-treated leaves, while levels of all other phyla were lower. Furthermore, lower abundances of Cyanobacteria, Unknown phyla and Planctomycets were detected on LAM-treated plants in comparison to H_2O-treated plants. At T1, sizes of bacterial phyla were comparable on H_2O-, NB- and LAM-treated leaves. Considering the proportions of bacterial phyla at the two time points, the levels of Actinobacteria, Cyanobacteria, Acidobacteria, Unknown phyla and Planctomycetes on H_2O-treated plants were reduced from T0 to T1. The relative abundance of dominant phyla was comparable on NB- and LAM-treated leaves at the two time points, except for the reduction of Actinobacteria on LAM-treated leaves from T0 to T1.

In Exp 2, H_2O application on grapevine leaves did not influence phyla proportions (Figure S7B). On leaves collected at T0, phyla abundances were similar on NB- and H_2O-treated plants, and the Cyanobacteria abundance was greater on LAM-treated plants in comparison to H_2O-treated plants. At T1, abundances of Proteobacteria and Firmicutes were lower and greater on NB-treated plants in comparison to H_2O-treated plants, respectively. Moreover, abundances of Proteobacteria decreased and those of Firmicutes increased from T0 to T1, and lower abundance of Actinobacteria and Cyanobacteria was detected at T0 with respect to T1 on LAM-treated plants.

Distribution of Fungal Phyla, Family and Genera among Experiments, Treatments and Time Points

Ascomycota was the most common fungal phylum detected, and only 2.3% of total fungal OTU (24 out of 1051) was attributed to Basidiomycota and Zygomycota. Of fungal reads, 85.5% was attributed to taxa at the family level (796 OTU), and 89.2% was assigned to a fungal genus (768 OTU); 34, 53 and 87 different fungal families, genera and species were identified in total, respectively (Table S7). The proportions of the 10, 7 and 15 dominant families (Figure S9), genera (Figure S10) and species (Figure S11) were homogeneous between the two experiments, and they were only slightly affected by treatments and time points.

The relative abundance of Arthrodermataceae decreased on UNT, LAM- and NB-treated plants in comparison to H_2O-treated plants at T0 in Exp 1, while comparable abundances were observed for the other dominant families among treatments (Figure S9A). At T1, the presence of Agaricaceae was greater on LAM-treated plants in comparison to H_2O-treated plants and comparable abundances were observed for all fungal families between NB- and H_2O-treated plants. Considering the proportions of fungal families at the two time points, abundances of Agaricaceae increased on H_2O- and LAM-treated plants from T0 to T1, while those of Arthrodermataceae decreased on H_2O-treated plants from T0 to T1. In Exp 2, H_2O treatment partially influenced family proportions at T0, and the relative abundance of Microascaceae and

Erysiphaceae decreased compared to UNT plants (Figure S9B). Relative abundances of fungal families were comparable on NB- and H_2O-treated plants, and the presence of Apiosporaceae increased on LAM-treated plants at T0 and T1. Considering proportions of fungal families at two different time points, only the abundance of Onygenaceae and Arthrodermataceae changed on NB- and LAM-treated plants from T0 to T1, respectively.

In Exp 1, relative abundances of dominant fungal genera were generally comparable on H_2O-treated and UNT plants, only the presence of *Peniciullium* spp. was decreased by H_2O treatment (Figure S10A). Percentages of fungal genera were comparable for H_2O-, NB- and LAM-treated plants at T0, while the presence of *Hansfordia* spp. was greater on LAM-treated plants in comparison to H_2O- and NB-treated plants at T1. Relative abundance of bacterial genera was comparable on H_2O-, NB- and LAM-treated plants at the two time points, except for an increase in *Hanfordia* levels on LAM-treated plants from T0 to T1. In Exp 2, H_2O application did not affect genera proportions at T0 (Figure S10B). Relative abundances of fungal genera were comparable on NB- and H_2O-treated plants at T0 and T1. Comparing H_2O- and LAM-treated plants, the presence of *Aspergillus* decreased at T0, whereas that of *Arthrinium* spp. increased at T0 and T1. Moreover, abundances of *Chrysosporium* spp. and *Hansfordia* spp. increased on NB- and LAM-treated leaves from T0 to T1, respectively.
