# Supplementary material for: Leaf Treatments with a Protein-Based Resistance Inducer Partially Modify Phyllosphere Microbial Communities of Grapevine
Source: Front Plant Sci. 2016 Jul 19;7:1053. doi: 10.3389/fpls.2016.01053 (PMC4949236; doi:10.3389/fpls.2016.01053)
Supplement: Supplementary file 11 [file Image1.PDF]

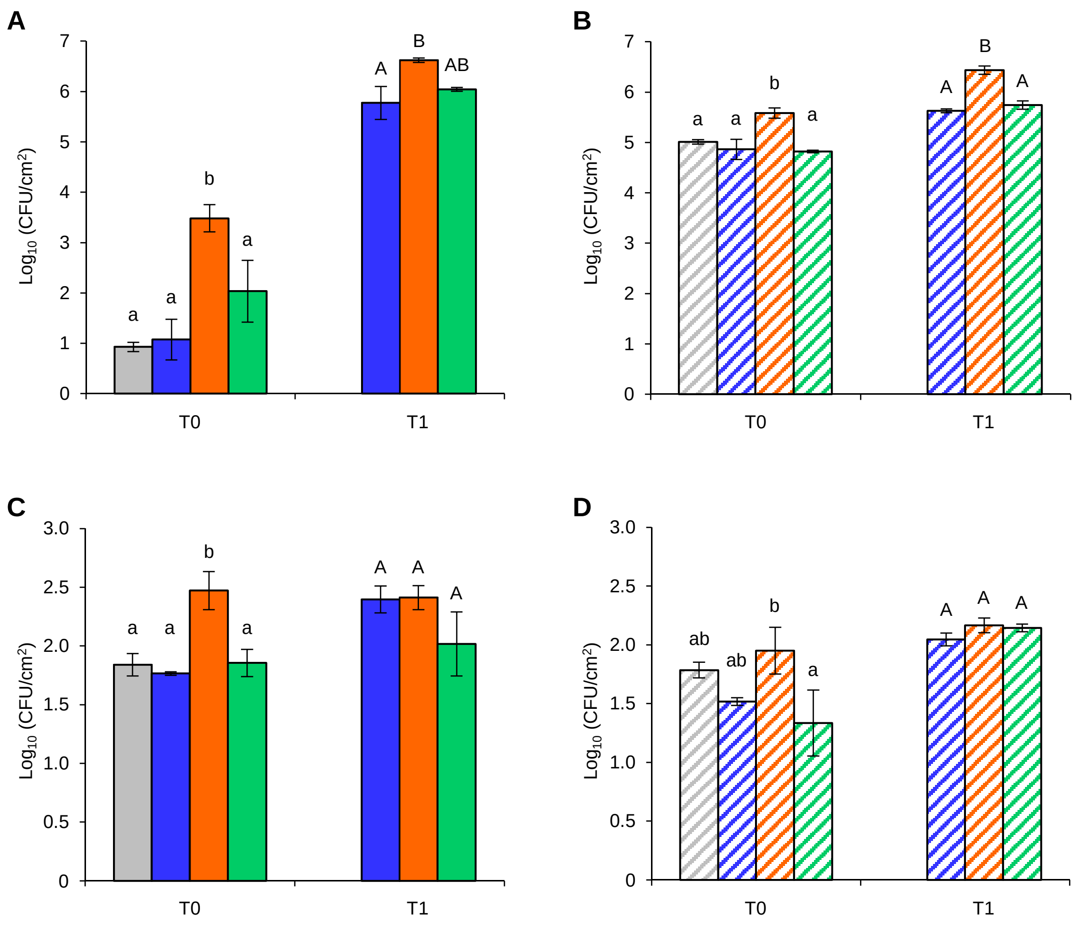

FIGURE S1 | Assessment of culturable microorganisms of grapevine leaves. Colony forming units (CFU) of bacteria (A, B) and fungi (C, D) per unit of grapevine leaf area (cm^2) were assessed for untreated plants (grey), and plants treated with water (blue), nutrient broth (orange) or laminarin (green) collected just before (T0) and one day after (T1) *Plasmopara viticola* in the experiment 1 (A, C) and experiment 2 (B, D), by plating method on selective media. Mean Log_{10} (CFU/cm^2) values and standard errors from three replicates (each as a pool of two plants) are presented for each sample. Different lowercase and uppercase letters indicate significant differences at T0 and T1 according to Fisher's test (α = 0.05), respectively.
