# Supplementary material for: Leaf Treatments with a Protein-Based Resistance Inducer Partially Modify Phyllosphere Microbial Communities of Grapevine
Source: Front Plant Sci. 2016 Jul 19;7:1053. doi: 10.3389/fpls.2016.01053 (PMC4949236; doi:10.3389/fpls.2016.01053)
Supplement: Supplementary file 12 [file Image2.PDF]

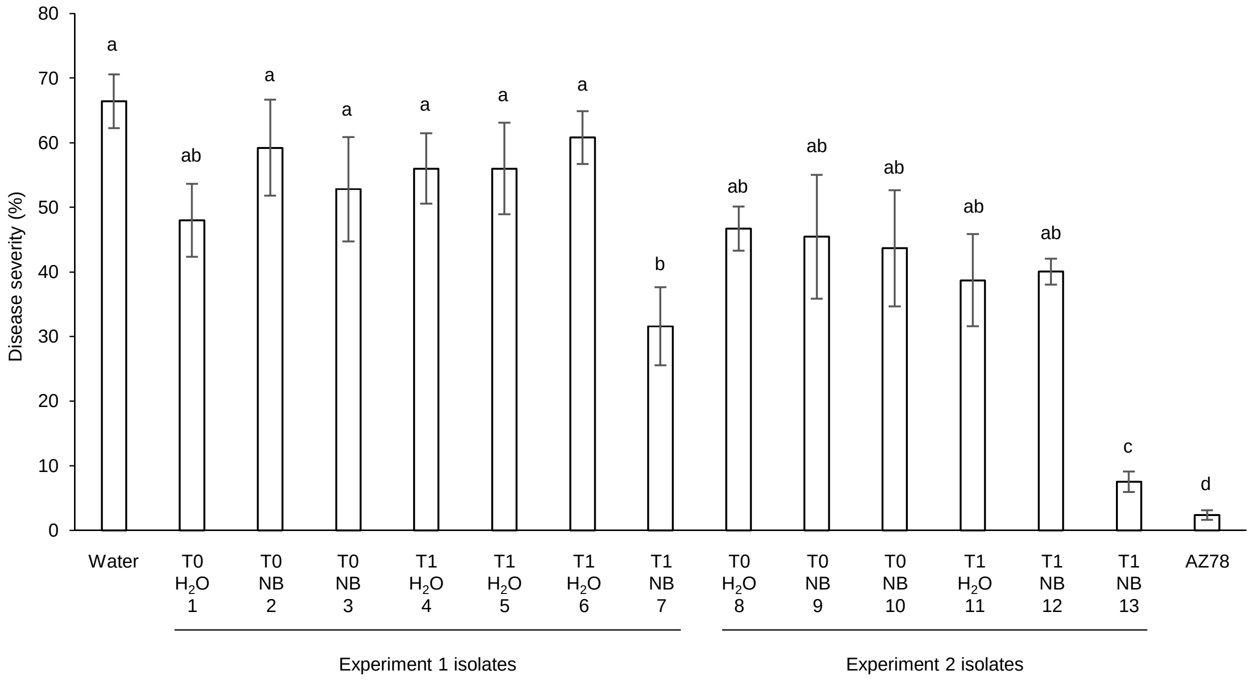

FIGURE S2 | Effect of culturable bacterial isolates against *Plasmopara viticola*. Seven and six bacterial isolates were randomly selected for experiment 1 and experiment 2, respectively, and tested against *P. viticola* on surface-sterilized leaf disks. Bacterial codes indicate time points (T0 or T1) and treatments (H_2O or NB) of leaf samples from where each bacteria was isolated, and a progressive numerical code assigned to the representative isolates. The pure *P. viticola* suspension (water) and the biocontrol strain *Lysobacter capsici* AZ78 (AZ78) were used as controls and disease severity was assessed as percentage of disc area covered by *P. viticola* sporulation seven days after inoculation. Mean severity and standard error values of five replicates (five dishes with five leaf disks each) of one representative experiment are reported for each treatment. Different letters indicate significant differences according to Fisher's test ($\alpha = 0.05$). Isolates with the greatest efficacy corresponded to a *Pseudomonas* sp. (T1_NB_7 of Exp 1) and an *Enterobacter* sp. (T1_NB_13 of Exp 2).
