# Supplementary material for: Leaf Treatments with a Protein-Based Resistance Inducer Partially Modify Phyllosphere Microbial Communities of Grapevine
Source: Front Plant Sci. 2016 Jul 19;7:1053. doi: 10.3389/fpls.2016.01053 (PMC4949236; doi:10.3389/fpls.2016.01053)
Supplement: Supplementary file 13 [file Image3.PDF]

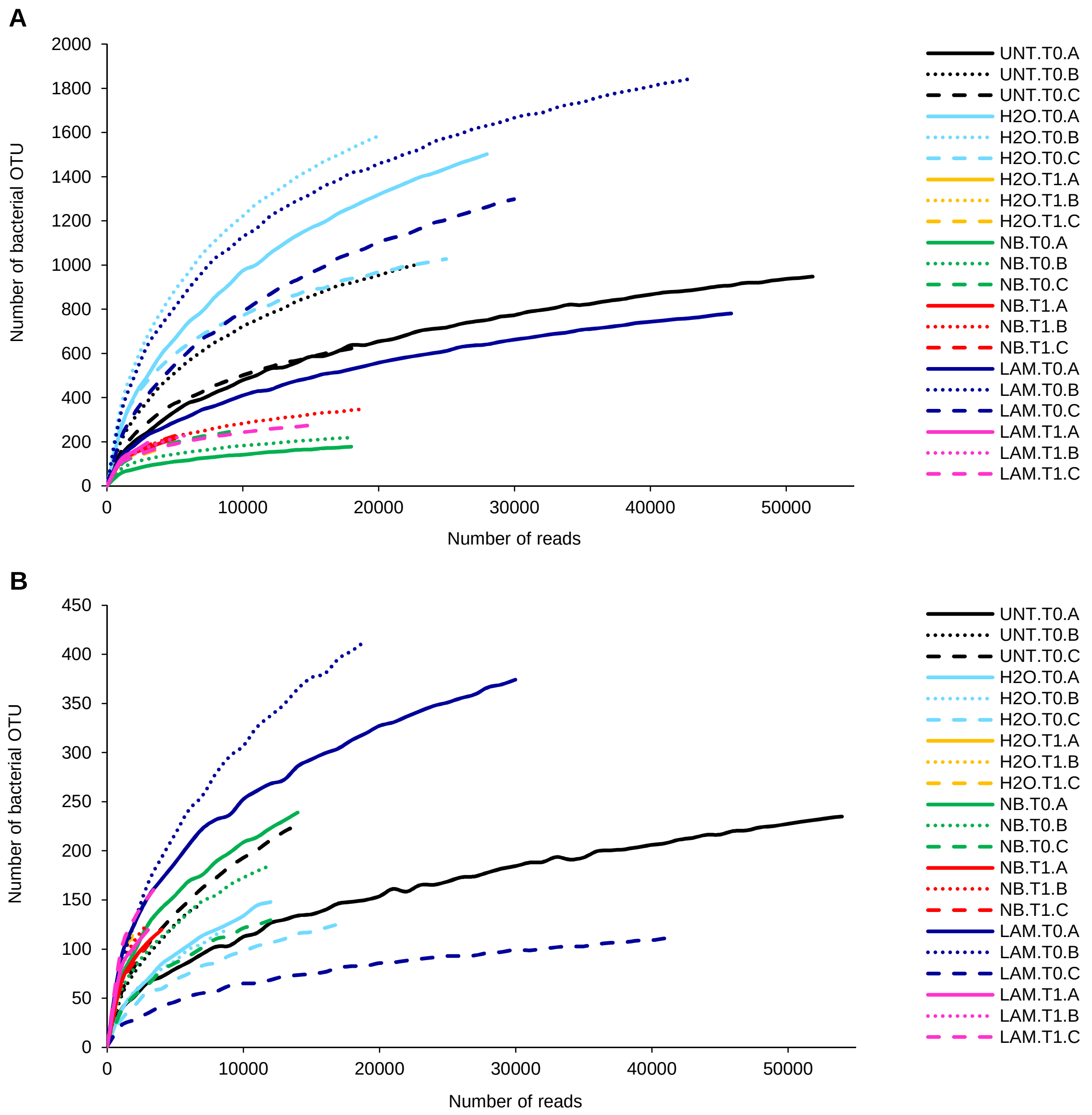

FIGURE S3 | Rarefaction curves of bacterial communities identified on grapevine leaves in the experiment 1 (A) and experiment 2 (B). Curves were obtained by random resampling without replacement using QIIME, for samples collected from untreated plants (UNT), and plants treated with water (H_2O), nutrient broth (NB) or laminarin (LAM) collected just before (T0) and one day after (T1) *Plasmopara viticola* inoculation. Three replicates (each as a pool of two plants) were analyzed for each treatment and each time point (replicate A: solid lines; B: dotted lines; C: dashed lines).
