# Supplementary material for: Leaf Treatments with a Protein-Based Resistance Inducer Partially Modify Phyllosphere Microbial Communities of Grapevine
Source: Front Plant Sci. 2016 Jul 19;7:1053. doi: 10.3389/fpls.2016.01053 (PMC4949236; doi:10.3389/fpls.2016.01053)
Supplement: Supplementary file 15 [file Image5.PDF]

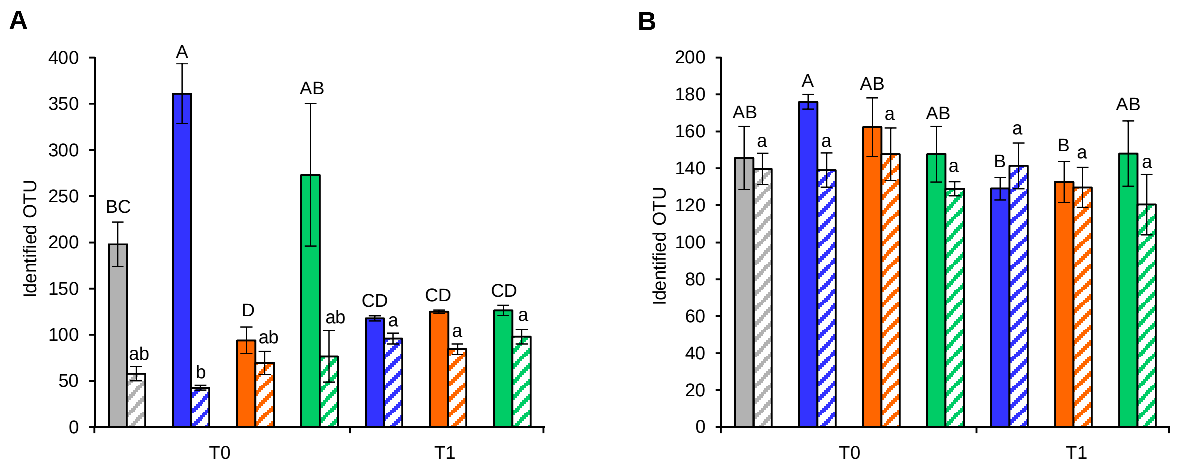


**FIGURE S5 | Richness of bacterial (A) and fungal (B) populations on grapevine leaves.** Operational taxonomic units (OTU) were determined for untreated plants (grey), and plants treated with water (blue), nutrient broth (orange) or laminarin (green) collected just before (T0) and one day after (T1) *Plasmopara viticola* inoculation, and normalized to the lowest number of quality filtered reads in experiment 1 (solid bars) and experiment 2 (striped bars). Mean and standard error values of three replicates (each as a pool of two plants) were analyzed for each treatment and time point. Different uppercase and lowercase letters indicate significant differences of experiment 1 and experiment 2 according to Fisher's test ($\alpha = 0.05$), respectively.
