# Supplementary material for: Leaf Treatments with a Protein-Based Resistance Inducer Partially Modify Phyllosphere Microbial Communities of Grapevine
Source: Front Plant Sci. 2016 Jul 19;7:1053. doi: 10.3389/fpls.2016.01053 (PMC4949236; doi:10.3389/fpls.2016.01053)
Supplement: Supplementary file 16 [file Image6.PDF]

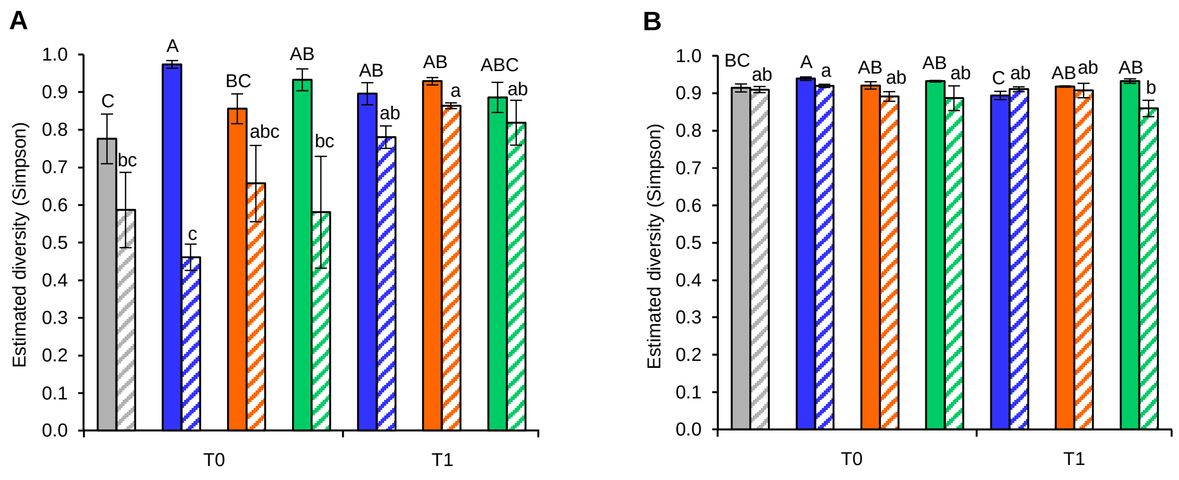


**FIGURE S6 | Diversity of bacterial (A) and fungal (B) populations on grapevine leaves.** The Simpson index was determined for untreated plants (grey), and plants treated with water (blue), nutrient broth (orange) and laminarin (green) collected just before (T0) and one day after (T1) *Plasmopara viticola* inoculation, and normalized to the lowest number of quality filtered reads in experiment 1 (solid bars) and experiment 2 (striped bars). Mean and standard error values of three replicates (each as a pool of two plants) were analyzed for each treatment and time point. Different uppercase and lowercase letters indicate significant differences of experiment 1 and experiment 2 according to Fisher's test ($\alpha$ = 0.05), respectively.
