# Supplementary material for: Leaf Treatments with a Protein-Based Resistance Inducer Partially Modify Phyllosphere Microbial Communities of Grapevine
Source: Front Plant Sci. 2016 Jul 19;7:1053. doi: 10.3389/fpls.2016.01053 (PMC4949236; doi:10.3389/fpls.2016.01053)
Supplement: Supplementary file 18 [file Image8.PDF]

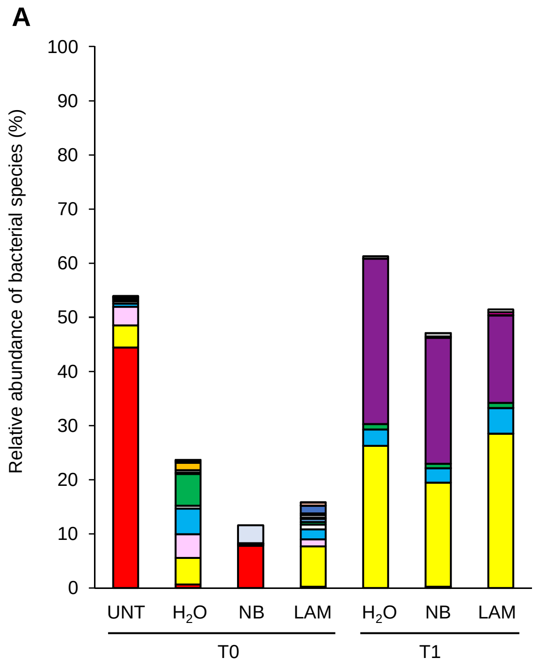

Species	UNT (T0)	H_2O (T0)	NB (T0)	LAM (T0)	H_2O (T1)	NB (T1)	LAM (T1)
Erwinia soli	b	b	b	b	a	a	a
Variovorax paradoxus	b	b	b	a	b	b	b
Pseudomonas alcaligenes	b	b	a	b	b	b	b
Acinetobacter rhizosphaerae	ab	ab	b	b	ab	ab	a
Microbacterium chocolatum	b	ab	b	a	b	b	b
Enhydrobacter aerosaccus	b	a	b	ab	b	b	b
Saccharopolyspora hirsuta	a	a	b	a	b	b	b
Acinetobacter schindleri	b	b	b	b	a	a	a
Escherichia coli	ab	a	b	a	b	b	b
Enterobacter cloacae	b	a	b	ab	ab	ab	ab
Desulfosporosinus meridiei	a	a	b	a	b	b	b
Pseudomonas viridiflava	cd	a	d	bc	ab	ab	ab
Pseudomonas veronii	a	a	b	ab	b	b	b
Pantoea agglomerans	c	c	c	bc	a	ab	a
Serratia marcescens	a	c	b	c	c	c	c

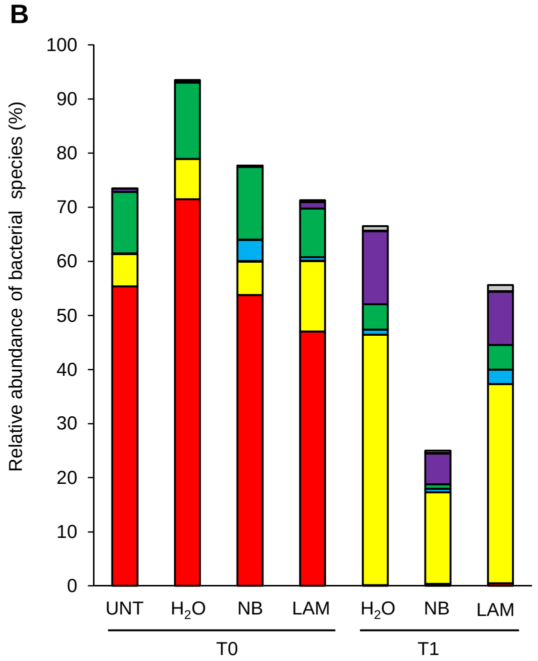

Species	UNT (T0)	H_2O (T0)	NB (T0)	LAM (T0)	H_2O (T1)	NB (T1)	LAM (T1)
Erwinia soli	bc	c	c	bc	a	ab	a
Variovorax paradoxus							
Pseudomonas alcaligenes							
Acinetobacter rhizosphaerae	b	ab	ab	b	ab	a	a
Microbacterium chocolatum							
Enhydrobacter aerosaccus							
Saccharopolyspora hirsuta							
Acinetobacter schindleri	c	c	c	c	a	b	ab
Escherichia coli							
Enterobacter cloacae	ab	a	ab	abc	abc	c	bc
Desulfosporosinus meridiei							
Pseudomonas viridiflava	b	b	a	b	ab	ab	a
Pseudomonas veronii	b	b	a	ab	b	b	b
Pantoea agglomerans	c	c	c	c	a	bc	ab
Serratia marcescens	a	a	a	a	b	b	b

FIGURE S8 | Relative abundance of the dominant (more than 0.5 % of relative abundance in at least one sample) bacterial species on grapevine leaves. Percentages of relative abundance were determined for leaves of untreated plants (UNT), and plants treated with water (H_2O), nutrient broth (NB) or laminarin (LAM) collected just before (T0) and one day after (T1) *Plasmopara viticola* inoculation in the experiment 1 (A) and experiment 2 (B). Mean and standard error values of three replicates (each as a pool of two plants) were analyzed for each treatment and time point. For each taxon, the intensity of the color gradient and letters reported in the table indicate significant differences among treatments and time points according to Fisher's test (α = 0.05).
