# Supplementary material for: Leaf Treatments with a Protein-Based Resistance Inducer Partially Modify Phyllosphere Microbial Communities of Grapevine
Source: Front Plant Sci. 2016 Jul 19;7:1053. doi: 10.3389/fpls.2016.01053 (PMC4949236; doi:10.3389/fpls.2016.01053)
Supplement: Supplementary file 20 [file Image10.PDF]

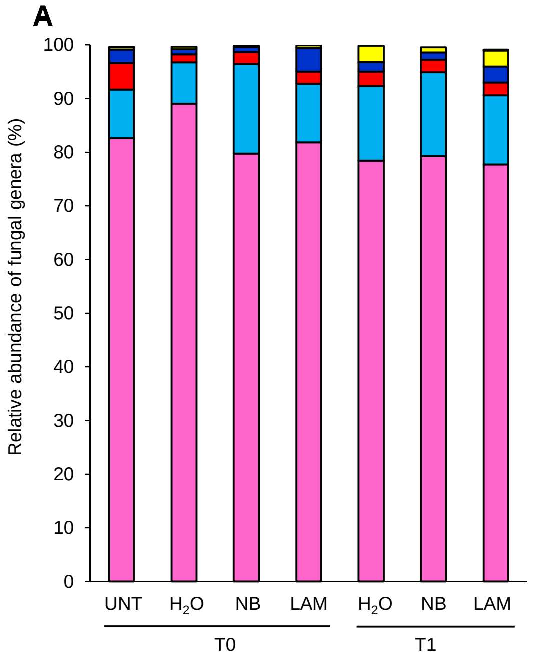

	T0 UNT	T0 H_2O	T0 NB	T0 LAM	T1 H_2O	T1 NB	T1 LAM
Chrysosporium							
Hansfordia	ab	b	b	b	b	b	a
Arthrinium	c	bc	abc	abc	abc	ab	a
Acremonium							
Penicillium	a	b	ab	ab	ab	ab	ab
Unknown							
Aspergillus							

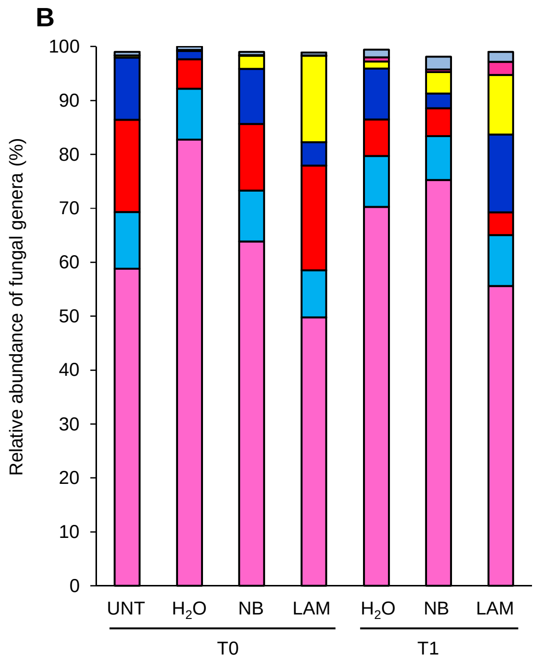

	T0 UNT	T0 H_2O	T0 NB	T0 LAM	T1 H_2O	T1 NB	T1 LAM
Chrysosporium	ab	ab	b	ab	ab	a	ab
Hansfordia	b	b	b	b	ab	ab	a
Arthrinium	bc	c	bc	a	bc	b	a
Acremonium							
Penicillium							
Unknown							
Aspergillus	ab	a	ab	b	ab	ab	ab

FIGURE S10 | Relative abundance of the dominant (more than 2 % of relative abundance in at least one sample) fungal genera on grapevine leaves. Percentages of relative abundance were determined for leaves of untreated plants (UNT), and plants treated with water (H_2O), nutrient broth (NB) or laminarin (LAM) collected just before (T0) and one day after (T1) *Plasmopara viticola* inoculation in the experiment 1 (A) and experiment 2 (B). Mean and standard error values of three replicates (each as a pool of two plants) were analyzed for each treatment and time point. For each taxon, the intensity of the color gradient and letters reported in the table indicate significant differences among treatments and time points according to Fisher's test ($\alpha = 0.05$).
