# Supplementary material for: Leaf Treatments with a Protein-Based Resistance Inducer Partially Modify Phyllosphere Microbial Communities of Grapevine
Source: Front Plant Sci. 2016 Jul 19;7:1053. doi: 10.3389/fpls.2016.01053 (PMC4949236; doi:10.3389/fpls.2016.01053)
Supplement: Supplementary file 21 [file Image11.PDF]

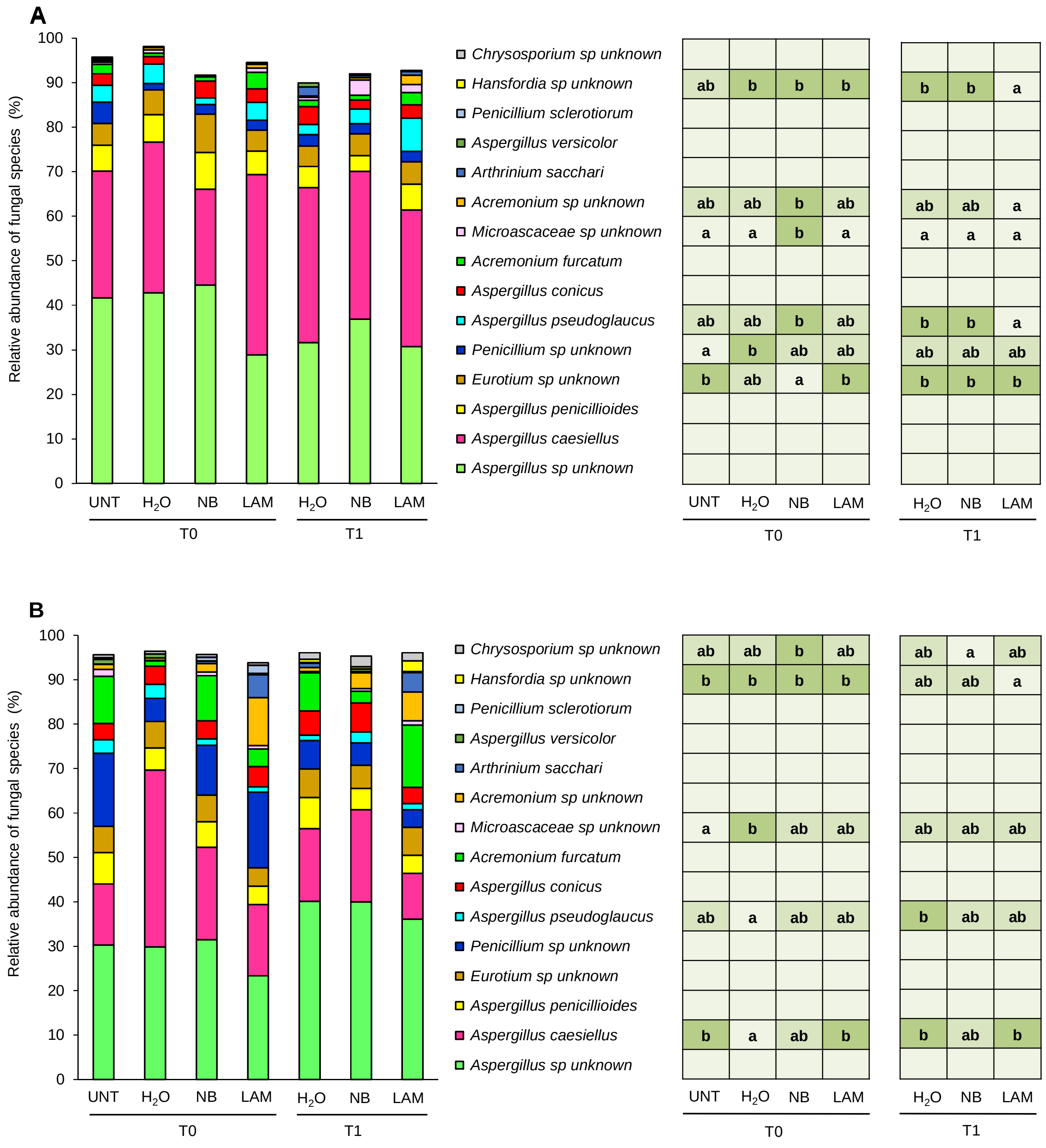

FIGURE S11 | Relative abundance of the dominant (more than 1 % of relative abundance in at least one sample) fungal species on grapevine leaves. Percentages of relative abundance were determined for leaves of untreated plants (UNT), and plants treated with water (H_2O), nutrient broth (NB) or laminarin (LAM) collected just before (T0) and one day after (T1) *Plasmopara viticola* inoculation in the experiment 1 (A) and experiment 2 (B). Mean and standard error values of three replicates (each as a pool of two plants) were analyzed for each treatment and time point. For each taxon, the intensity of the color gradient and letters reported in the table indicate significant differences among treatments and time points according to Fisher's test (α = 0.05).
